# Utilizing CMP-Sialic Acid Analogs to Unravel *Neisseria gonorrhoeae* Lipooligosaccharide-Mediated Complement Resistance and Design Novel Therapeutics

**DOI:** 10.1371/journal.ppat.1005290

**Published:** 2015-12-02

**Authors:** Sunita Gulati, Ian C. Schoenhofen, Dennis M. Whitfield, Andrew D. Cox, Jianjun Li, Frank St. Michael, Evgeny V. Vinogradov, Jacek Stupak, Bo Zheng, Makoto Ohnishi, Magnus Unemo, Lisa A. Lewis, Rachel E. Taylor, Corinna S. Landig, Sandra Diaz, George W. Reed, Ajit Varki, Peter A. Rice, Sanjay Ram

**Affiliations:** 1 Division of Infectious Diseases and Immunology, University of Massachusetts Medical School, Worcester, Massachusetts, United States of America; 2 Human Health Therapeutics Portfolio, National Research Council of Canada, Ottawa, Ontario, Canada; 3 National Institute of Infectious Diseases, Tokyo, Japan; 4 WHO Collaborating Centre for Gonorrhoea and Other STIs, Department of Laboratory Medicine, Microbiology, Örebro University Hospital, Örebro, Sweden; 5 Biomedical Sciences Graduate Program, Departments of Medicine and Cellular and Molecular Medicine, Glycobiology Research and Training Center, University of California, San Diego, La Jolla, California, United States of America; 6 Preventive and Behavioral Medicine, University of Massachusetts Medical School, Worcester, Massachusetts, United States of America; Université René Descartes, Faculté de Mîdecine, FRANCE

## Abstract

*Neisseria gonorrhoeae* deploys a novel immune evasion strategy wherein the lacto-N-neotetraose (LNnT) structure of lipooligosaccharide (LOS) is capped by the bacterial sialyltransferase, using host cytidine-5’-monophosphate (CMP)-activated forms of the nine-carbon nonulosonate (NulO) sugar *N*-acetyl-neuraminic acid (Neu5Ac), a sialic acid (Sia) abundant in humans. This allows evasion of complement-mediated killing by recruiting factor H (FH), an inhibitor of the alternative complement pathway, and by limiting classical pathway activation (“serum-resistance”). We utilized CMP salts of six additional natural or synthetic NulOs, Neu5Gc, Neu5Gc8Me, Neu5Ac9Ac, Neu5Ac9Az, legionaminic acid (Leg5Ac7Ac) and pseudaminic acid (Pse5Ac7Ac), to define structural requirements of Sia-mediated serum-resistance. While all NulOs except Pse5Ac7Ac were incorporated into the LNnT-LOS, only Neu5Gc incorporation yielded high-level serum-resistance and FH binding that was comparable to Neu5Ac, whereas Neu5Ac9Az and Leg5Ac7Ac incorporation left bacteria fully serum-sensitive and did not enhance FH binding. Neu5Ac9Ac and Neu5Gc8Me rendered bacteria resistant only to low serum concentrations. While serum-resistance mediated by Neu5Ac was associated with classical pathway inhibition (decreased IgG binding and C4 deposition), Leg5Ac7Ac and Neu5Ac9Az incorporation did not inhibit the classical pathway. Remarkably, CMP-Neu5Ac9Az and CMP-Leg5Ac7Ac each prevented serum-resistance despite a 100-fold molar excess of CMP-Neu5Ac in growth media. The concomitant presence of Leg5Ac7Ac and Neu5Ac on LOS resulted in uninhibited classical pathway activation. Surprisingly, despite near-maximal FH binding in this instance, the alternative pathway was not regulated and factor Bb remained associated with bacteria. Intravaginal administration of CMP-Leg5Ac7Ac to BALB/c mice infected with gonorrhea (including a multidrug-resistant isolate) reduced clearance times and infection burden. Bacteria recovered from CMP-Leg5Ac7Ac-treated mice were sensitive to human complement *ex vivo*, simulating *in vitro* findings. These data reveal critical roles for the Sia exocyclic side-chain in gonococcal serum-resistance. Such CMP-NulO analogs may provide a novel therapeutic strategy against the global threat of multidrug-resistant gonorrhea.

## Author Summary

*Neisseria gonorrhoeae*, the causative agent of the sexually transmitted infection gonorrhea, has developed widespread resistance to almost every conventional antibiotic currently in clinical use. Novel therapeutics are urgently needed against this pathogen. Gonococci have the capacity to scavenge CMP-*N*-acetyl-neuraminic acid (CMP-Neu5Ac, a CMP-activated 9-carbon sugar that is a member of the ‘sialic acid family’) from the host to ‘cap’ its lipooligosaccharide with Neu5Ac, which renders gonococci resistant to complement, a key arm of innate immune defenses. Here, we show that gonococci also utilize derivatives (or analogs) of CMP-Neu5Ac, which not only fail to render the bacteria resistant to complement, but also prevent complement inhibition mediated by the ‘physiologic’ human sialic acid donor, CMP-Neu5Ac. When administered intravaginally to mice, a representative analog significantly shortened the duration and burden of gonococcal infection. Thus, CMP-sialic acid analogs may represent promising preventive or therapeutic agents against multidrug-resistant gonorrhea that poses a global threat to public health.

## Introduction

Sialic acids (Sias) are a family of 9-carbon sugars (nonoses, or nonulosonates (NulOs)) expressed in the tissues of every vertebrate and some “higher” invertebrates [1]. Sias serve a wide variety of biological roles, including modulation of several aspects of immune function [2]. For example, cell surface-associated Sia regulates the immune system by inhibiting complement activation. Sheep erythrocytes are resistant to lysis by the human alternative pathway because surface Sias increase the affinity of factor H (FH; inhibitor of the alternative complement pathway) for C3b, that is deposited on these or other host cells or microbial surfaces upon activation of complement [3]. Recent work has shown that FH C-terminal domains 19 and 20 bound simultaneously to C3b and glycosaminoglycans or Sias on host cells, which served to inhibit the alternative pathway [4]. This was further confirmed by structural data that suggest a trimolecular complex of the two C-terminal FH domains, Sia and C3b [5]. Neuraminidase treatment of sheep erythrocytes removes cell surface Sias and reduces the affinity of FH for C3b, which permits complement activation and promotes hemolysis. Typically, FH binds vertebrate cell surfaces via Sias to allow preferential protection of host cells (i.e., reduce complement-mediated damage).

Many microbes express Sias and other unique microbial NulOs (e.g., legionaminic (Leg) and pseudaminic (Pse) acid) on their surfaces that contribute to pathogenesis in numerous ways including subverting complement activation, promoting biofilm formation and facilitating colonization [6]. Some pathogens such as *Neisseria gonorrhoeae (Ng)*, *Haemophilus influenzae*, *Histophilus somni* (*Haemophilus somnus*) and serogroup A *N*. *meningitidis* lack the ability to synthesize Sias, but scavenge these molecules (such as Neu5Ac or Neu5Gc, or the cytidine-monophospho (CMP)-activated form CMP-Neu5Ac) from the host. Other pathogens, for example, *Escherichia coli* K1, *Streptococcus agalactiae*, *N*. *meningitidis*, groups B, C, W and Y, *Campylobacter jejuni* and certain Leptospira, can synthesize their own nonulosonic acids such as Neu5Ac, Leg5Ac7Ac or Pse5Ac7Ac *de novo*. Sialylation of gonococcal lacto-N-neotetraose (LNnT; Galβ1-4GlcNAcβ1-3Gal-Glcβ1-4HepI) lipooligosaccharide (LOS) using CMP-Neu5Ac enhances resistance of *Ng* to complement-dependent killing by decreasing binding of IgG against select bacterial targets such as porin B (PorB) protein [7], which attenuates the classical pathway. LNnT LOS sialylation with Neu5Ac also enhances FH binding, which results in inhibition of the alternative pathway [8].

The purpose of this study was to use CMP-NulOs to define the structural basis of Neu5Ac-mediated complement inhibition by gonococci. CMP-NulO analogs that serve as substrates for gonococcal LOS sialyltransferase (Lst) and result in NulO modified LOS, may prevent Neu5Ac-mediated serum resistance. This could translate into a novel therapeutic approach to combat infections caused by *Ng*, a microorganism that has developed resistance to almost every conventional antibiotic.

## Results

### Substrate specificity of gonococcal lipooligosaccharide sialyltransferase

**Fig 1:**
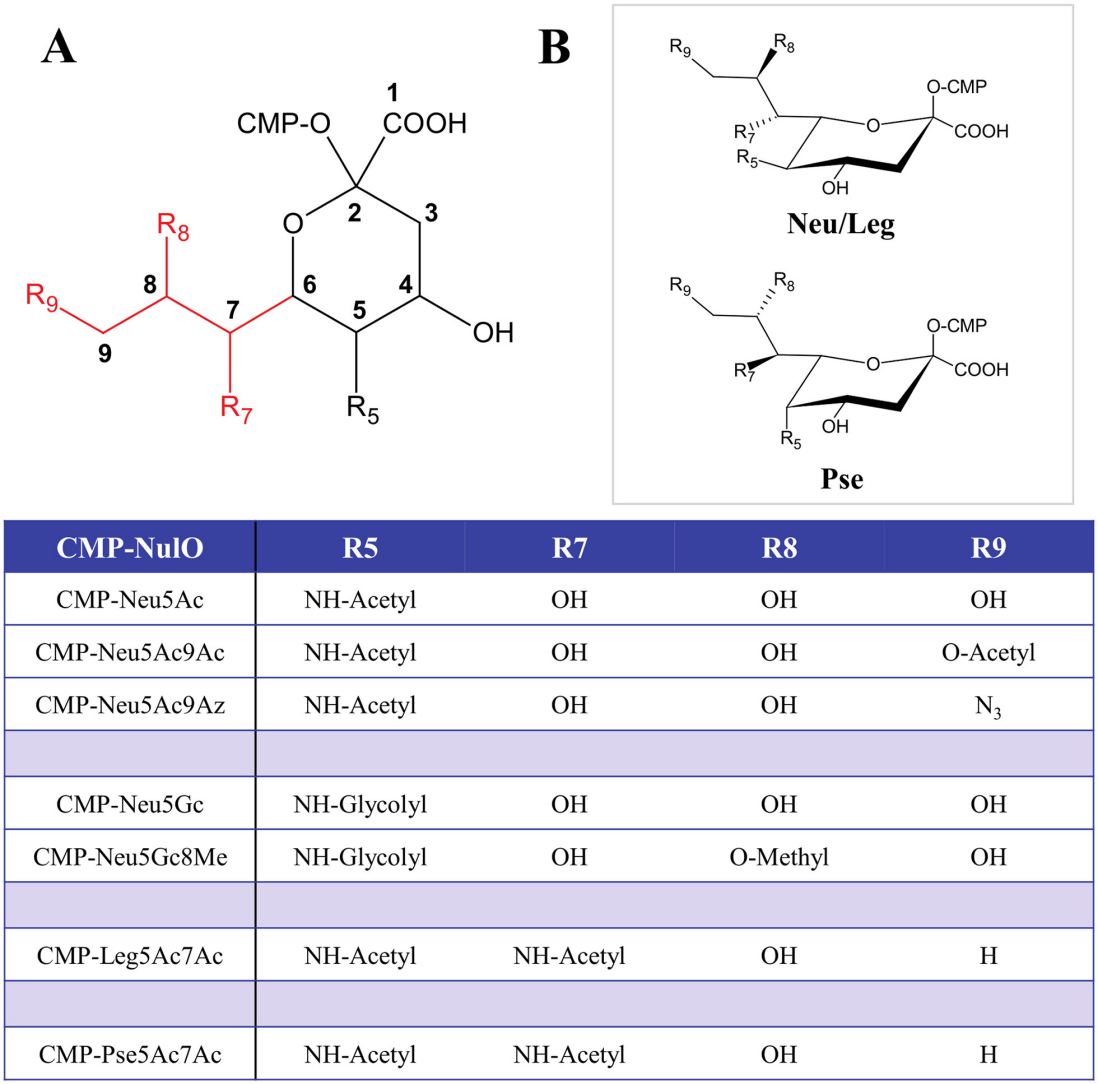
Structures of the CMP-nonulosonate (CMP-NulO) analogs used in this study. **A** General chemical structure, and **B** ^2^C_5_ chair chemical structure of CMP-nonulosonates used. All NulO sugars except for pseudaminic acid (Pse5Ac7Ac) have the same stereochemistry (D-*glycero*-D-*galacto* configuration), where Pse has an L-*glycero*-L-*manno* configuration, differing stereochemically at carbons 5, 7 and 8. For reference, the nine carbon atoms of the NulOs are numbered in **A**, where the NulO exocyclic moiety is highlighted in red.

Humans, unlike other old-world primates, lack a functional CMP-Neu5Ac hydroxylase enzyme (CMAH) and cannot convert CMP-Neu5Ac to the C5 glycolyl (Gc) derivative, CMP-Neu5Gc. In addition to CMP-Neu5Gc (relevant for the mouse model of gonorrhea), we also included other analogs with additional structural variations at carbons 7, 8 and 9, the exocyclic moiety of NulOs. Fig 1 illustrates the CMP-NulOs selected and prepared for this study. In summary, the analogs synthesized include two C9 modified CMP-Neu5Ac analogs (CMP-Neu5Ac9Ac, CMP-Neu5Ac9Az), a C8 modified CMP-Neu5Gc analog (CMP-Neu5Gc8Me), and two C7/C9 modified CMP-Neu5Ac analogs (CMP-Leg5Ac7Ac, CMP-Pse5Ac7Ac) were synthesized. See Materials and Methods and also S1 and S2 Tables and S1 Fig for further details of synthesis/characterization. All of the CMP-NulO analogs except CMP-Pse5Ac7Ac exhibit the same absolute configuration as CMP-Neu5Ac, making them candidate substrates for gonococcal LOS sialyltransferase (Lst).

**Fig 2:**
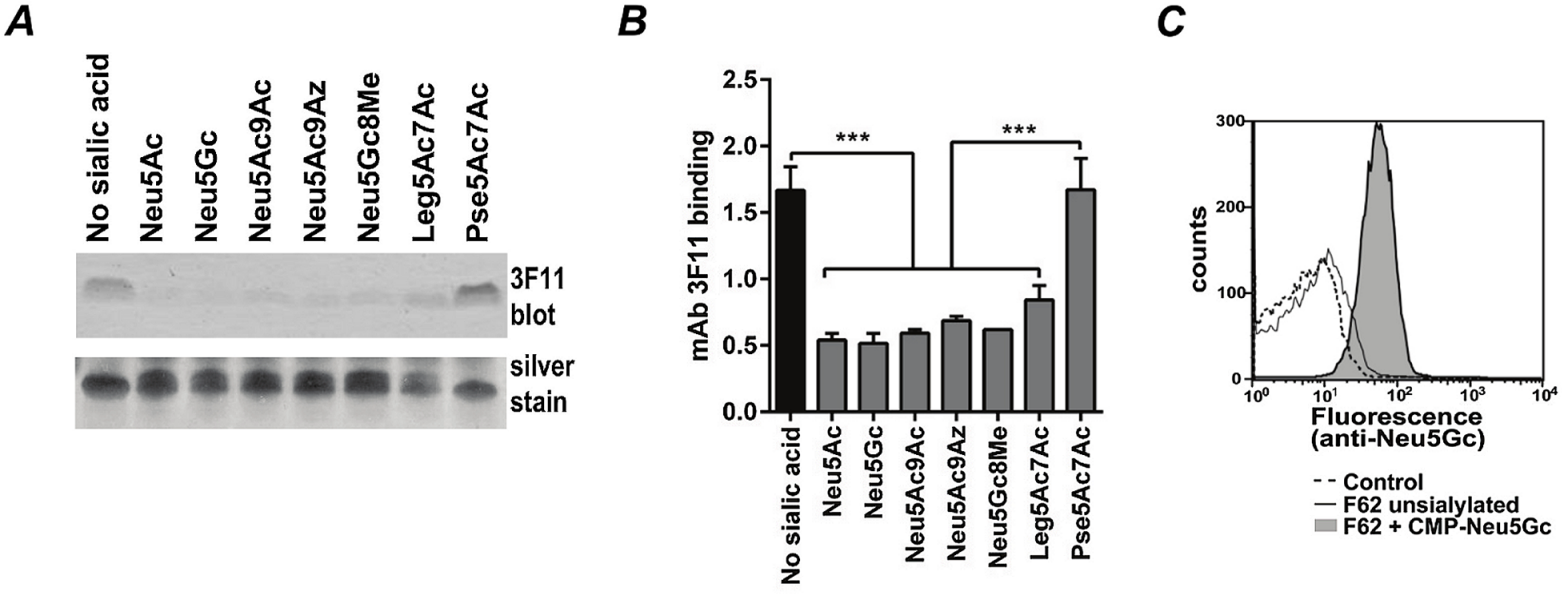
Substrate specificity of gonococcal LOS sialyltransferase (Lst). ***A***. *N*. *gonorrhoeae (Ng)* strain F62 ΔlgtD was grown in gonococcal media alone (‘No sialic acid’) or in media supplemented with CMP salts of the indicated sialic acid (Sia), each at a concentration of 20 μg/ml (30 μM), for 2 h at 37°C. Bacteria were washed, pellets treated with protease K, lysed in 4× LDS buffer and lysates were separated on 12% Bis-Tris gels using MES running buffer. LOS was transferred to a PVDF membrane and probed with monoclonal antibody (mAb) 3F11 that recognizes the lacto-*N*-neotetraose only in the non-sialylated state; the addition of a Sia residue abrogates mAb 3F11 binding (upper panel). LOS was also visualized by silver staining following electrophoresis on 16.5% tricine gels (lower panel). ***B***. Incorporation of NulO by LNnT LOS was assessed using whole cell ELISA with mAb 3F11, *** P<0.001. ***C***. Wild-type *Ng* F62 was grown with (shaded) or without (solid line) 30 μM CMP-Neu5Gc for 3 hours and analyzed by flow cytometry using a polyclonal Neu5Gc-specific chicken IgY Ab followed by a FITC conjugated donkey anti-chicken IgY secondary Ab. As a negative control, wild-type *Ng* F62 was grown in CMP-Neu5Gc as above and incubated with the secondary Ab only (dashed line).

Gonococcal Lst adds a terminal Neu5Ac residue to LNnT LOS when CMP-Neu5Ac is supplied exogenously (gonococci are unable to synthesize CMP-Neu5Ac) [9]. To define the substrate specificity of gonococcal Lst, we used an isogenic LOS glycosyltransferase D (*lgtD*) deletion mutant of gonococcal strain F62, called *Ng* F62 ΔlgtD in subsequent *in vitro* experiments. *LgtD* is a phase-variable gene product that adds a terminal GalNAc residue to HepI LNnT [10]; ‘capping’ LNnT with GalNAc will prevent LOS sialylation. Thus, deleting *lgtD* permits more homogenous expression of LNnT and uniform sialylation. *Ng* F62 ΔlgtD was grown in media alone (unsialylated) or in media containing either CMP-Neu5Ac or one of the other six CMP-NulOs (listed in the Table 1 and Fig 1), each at a concentration of ~30 μM (20 μg/ml). Following incubation for 2 h at 37°C, bacterial LOS was examined by western blotting using monoclonal antibody (mAb) 3F11, which binds to the terminal lactosamine residue of LNnT; any extension beyond the terminal Gal (for example with a NulO) will abrogate mAb 3F11 binding. As shown in Fig 2A, growth in media containing CMP salts of Neu5Ac, Neu5Gc, Neu5Ac9Ac, Neu5Ac9Az, Neu5Gc8Me and Leg5Ac7Ac resulted in decreased binding of mAb 3F11. This suggests that these CMP-NulOs served as substrates for gonococcal Lst in the context of live bacteria and the respective NulOs are incorporated into LNnT. Only Pse5Ac7Ac was not incorporated into LNnT LOS. CMP-Pse5Ac7Ac, differs from the other CMP-NulOs stereochemically at C5, C7 and C8, and was not anticipated to be utilized by gonococcal Lst. Consistent with decreased mAb 3F11 binding and addition of a NulO residue, silver staining of LOS showed the appearance of a second, slower migrating band in the 6 inside lanes (Fig 2A). Whole cell ELISA with mAb 3F11 confirmed results of western blotting (Fig 2B). Direct measurement of NulO incorporation into wild-type *Ng* F62 was shown for Neu5Gc using chicken polyclonal IgY Ab that specifically recognizes Neu5Gc (Fig 2C). This method directly demonstrates the presence of Neu5Gc on the bacterial surface. Finally, mass spectrometric analysis of LOS from bacteria grown in CMP-NulOs confirmed addition of the respective NulO onto LOS (S3 Table).

**Table 1:** Summary of nonulosonate (NulO) incorporation by *N*. *gonorrhoeae* lipooligosaccharide and key functional consequences.

NulO	Incorporation into LOS	Serum resistance	FH binding	IgG binding	C3 / C4 deposition
**None (unsialylated)**	−	none	−	+++	+++
**Neu5Ac**	Yes	high ^A^	+++	+ / −	+ / −
**Neu5Gc**	Yes	high	+++	ND ^B^	ND
**Neu5Ac9Ac**	Yes	low ^C^	+	+	++
**Neu5Ac9Az**	Yes	None	−	++	+++
**Neu5Gc8Me**	Yes	low ^C^	−	+	++
**Leg5Ac7Ac**	Yes	none	−	+++	+++
**Pse5Ac7Ac**	No	none	−	ND	ND

^A^ >100% survival in 10% serum

^B^ ND, not done

^C^ >100% survival in 3.3% serum and <10% survival in 10% serum

### Serum resistance mediated by incorporation of NulOs

**Fig 3:**
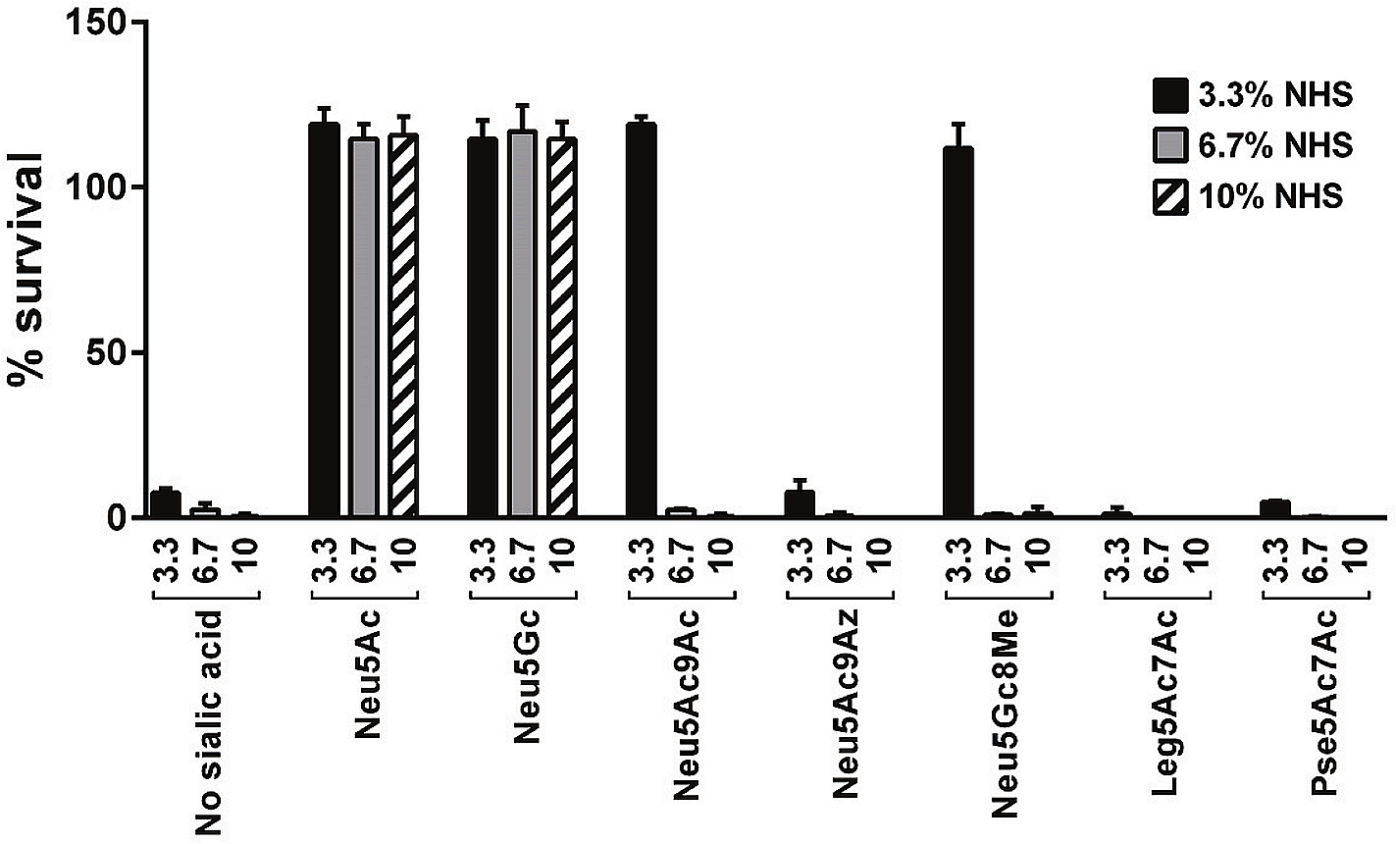
Select sialic acid (Sia) analogs enhance gonococcal serum resistance. *N*. *gonorrhoeae* (*Ng)* F62 ΔlgtD was grown in media alone (no CMP-Sia added), or media that contained 20 μg/ml (~30 μM) of each of the indicated CMP-Sias (NulOs). Resistance of bacteria to complement-dependent killing in the presence of 3.3%, 6.7% or 10% normal human serum (NHS) was measured in serum bactericidal assays. The mean (SD) of two independent experiments is shown. A survival greater than 100% indicates growth of bacteria.

The addition of a terminal Neu5Ac residue to the LNnT LOS of *Ng*, as occurs *in vivo* or following the addition of CMP-Neu5Ac to growth media, results in resistance to complement-dependent killing [11]. We next determined the effects of incorporation of the five structural analogs of Neu5Ac on the ability of *Ng* F62 ΔlgtD ability to resist complement-dependent killing by normal human serum at concentrations of 10%, 6.7% or 3.3%. Bacteria were grown either in media alone, or media supplemented with 30 μM (~20 μg/ml) of each of the CMP-NulOs. As shown in Fig 3, only CMP-Neu5Ac (serum-resistant control) and CMP-Neu5Gc conferred full (>100%) survival at serum concentrations of 10%. Neu5Ac9Ac and Neu5Gc8Me incorporation conferred >100% survival only in 3.3% serum, but did not protect bacteria (<10% survival) when serum concentrations were raised to 6.7%. The addition of Neu5Ac9Az and Leg5Ac7Ac to LOS did not increase bacterial survival at any serum concentration tested. As expected, Pse5Ac7Ac, which does not incorporate into LOS, did not affect serum resistance.

### FH binding and complement activation mediated by incorporation of NulOs

**Fig 4:**
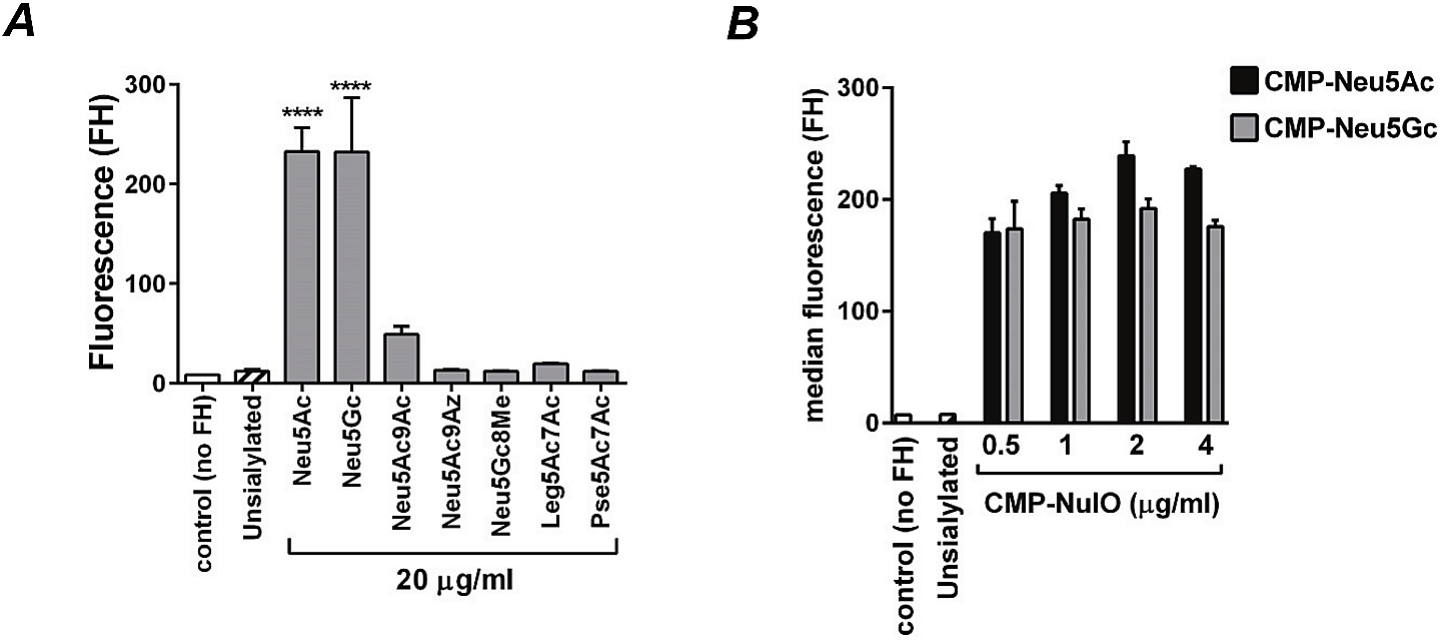
Factor H (FH) binding when sialic acid (Sia) analogs (NulOs) were substituted into *N*. *gonorrhoeae* (*Ng)* lipooligosaccharide (LOS). ***A***. *Ng* F62 ΔlgtD was grown for 2 h in media alone, or media supplemented with 20 μg/ml (~30 μM) of each of the indicated CMP-sialic acids (NulOs). Binding of bacteria to FH (10 μg/ml) was measured by flow cytometry using anti-FH mAb 90X. The control (open bar) indicates reaction mixtures that lacked FH. The median fluorescence was recorded for each reaction and the Y-axis shows the mean fluorescence (SD) of FH binding of 2 independent experiments. The mean (SD) of 2 independent repeat experiments is shown. ***B***. FH binding to *Ng* F62 ΔlgtD grown in CMP-Neu5Ac and CMP-Neu5Gc at concentrations ranging from 0.5 μg/ml to 4 μg/ml. Bacteria were incubated with FH (1 μg/ml) and bound FH was detected as indicated above.

A schematic of the complement cascade is provided in S2 Fig. The addition of a terminal Neu5Ac residue to LNnT LOS enhances binding of the alternative pathway inhibitor, FH, and this contributes to the ability of sialylated gonococci to resist killing by complement [8]. We next examined FH binding to *Ng* F62 ΔlgtD grown in the presence of each of the CMP-NulOs (20 μg/ml each) (Fig 4; see S3 Fig for representative histograms). Maximal FH binding was seen with Neu5Ac and Neu5Gc, a modest level of FH binding was seen with Neu5Ac9Ac; the remaining NulOs did not enhance FH binding significantly above levels seen with the unsialylated strain. Further, modification of LOS with Neu5Ac or Neu5Gc yielded similar amounts of FH binding, even with low CMP-Sia concentrations ranging from 0.5 to 4 μg/ml (~0.75–~4 μM), with near maximal FH binding observed at the lowest concentration tested (Fig 4B).

We next examined binding of *Ng*-specific IgG and IgM present in normal human serum (NHS), and deposition of complement components C3, C4 and factor B (FB) on *Ng* F62 ΔlgtD grown in the presence of the following representative CMP-NulOs: Neu5Ac represented a NulO that bound high levels of FH and conferred high level serum resistance; Neu5Gc8Me did not bind FH and conferred low level serum resistance; Neu5Ac9Ac bound modest amounts of FH and conferred low levels of serum resistance and Neu5Ac9Az and Leg5Ac7Ac neither bound FH nor conferred serum resistance. *Ng* F62 ΔlgtD with an unmodified (unsialylated) LNnT LOS was used as the control serum sensitive strain. Experiments were carried out using serum concentrations of 3.3% and 10%; the 3.3% serum concentration discriminated serum-killing of the Neu5Ac9Az phenotype from serum—resistance of the Neu5Gc8Me and Neu5Ac9Ac phenotypes.

**Fig 5:**
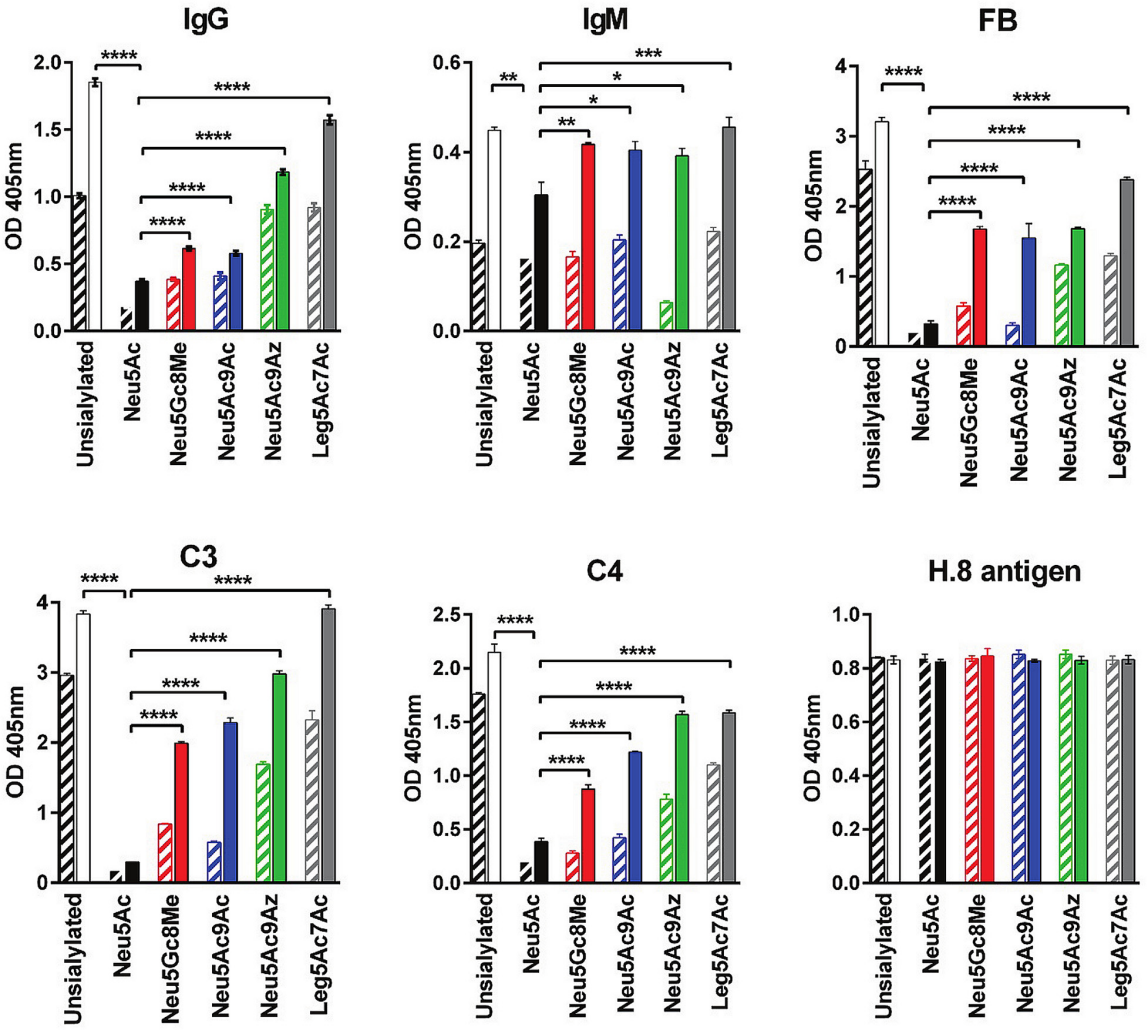
IgG, IgM, and complement components C3, C4 and FB deposition on *N*. *gonorrhoeae* (*Ng)* F62 that have incorporated each indicated sialic acid analog into lipooligosaccharide (LOS). *Ng* F62 ΔlgtD was grown in media alone (open bars), or media supplemented with 20 μg/ml (~30 μM) of each of the indicated CMP-Sias (NulOs). Bacteria were incubated with either 3.3% or 10% normal human serum (NHS) at 37°C for 10 min and IgG and IgM binding, and C3, C4 and factor B (FB) deposited on bacteria were measured by whole cell ELISA. Data with 3.3% and 10% NHS is shown using hatched and solid bars, respectively. mAb 2-8C-4-1 [62] that detects the Neisserial lipoprotein H.8 was similar across all groups and confirmed similar capture of bacteria in all wells (bottom right graph). Measurement of gonococcal H.8 lipoprotein antigen was performed to assess similarity of bacterial capture across microtiter wells. The mean (SD) of three observations is shown. Significance of differences in Ig or complement component binding/deposition using each analog vs, Neu5Ac is indicated for results that used 10% NHS only (for simplicity): *, P<0.05; **, P<0.01; ***, P<0.001; ****, P<0.0001 (ANOVA). Controls with bacteria alone yielded OD_405nm_ readings <0.1 (negative).

Consistent with previous studies [7,8,12], modification of LOS with Neu5Ac decreased IgG binding and resulted in maximal inhibition of complement (lowest C3, C4 and FB deposition) at both serum concentrations tested, while high levels of complement activation products were deposited on *Ng* F62 ΔlgtD (unsialylated) (Fig 5). LOS substitution with Neu5Ac significantly decreased IgG and IgM binding compared to all other CMP-NulOs, which could at least in part, contribute to decreased C4 deposition. While LOS modification with Neu5Ac9Ac and Neu5Gc8Me (both resistant only to 3.3%, but not 10% serum) yielded low levels of C3, C4 and FB deposition in 3.3% serum, only a modest decrease in deposition of these components was noted in 10% serum when compared with *Ng* F62 ΔlgtD (unsialylated) that was not likely to be sufficient to subvert killing by complement. Neu5Gc8Me and Neu5Ac9Ac substitution also reduced IgG binding, but not to the extent seen with Neu5Ac. The two analogs that left *Ng* F62 ΔlgtD fully serum sensitive, Neu5Ac9Az and Leg5Ac7Ac, yielded the highest levels of Ig binding and complement deposition among the analogs tested. A summary of CMP-NulOs utilized by Lst, Ab and complement component binding, and serum resistance associated with each of the tested CMP-NulOs is found in Table 1.

### Select NulO derivatives inhibit Neu5Ac-mediated serum resistance

**Fig 6:**
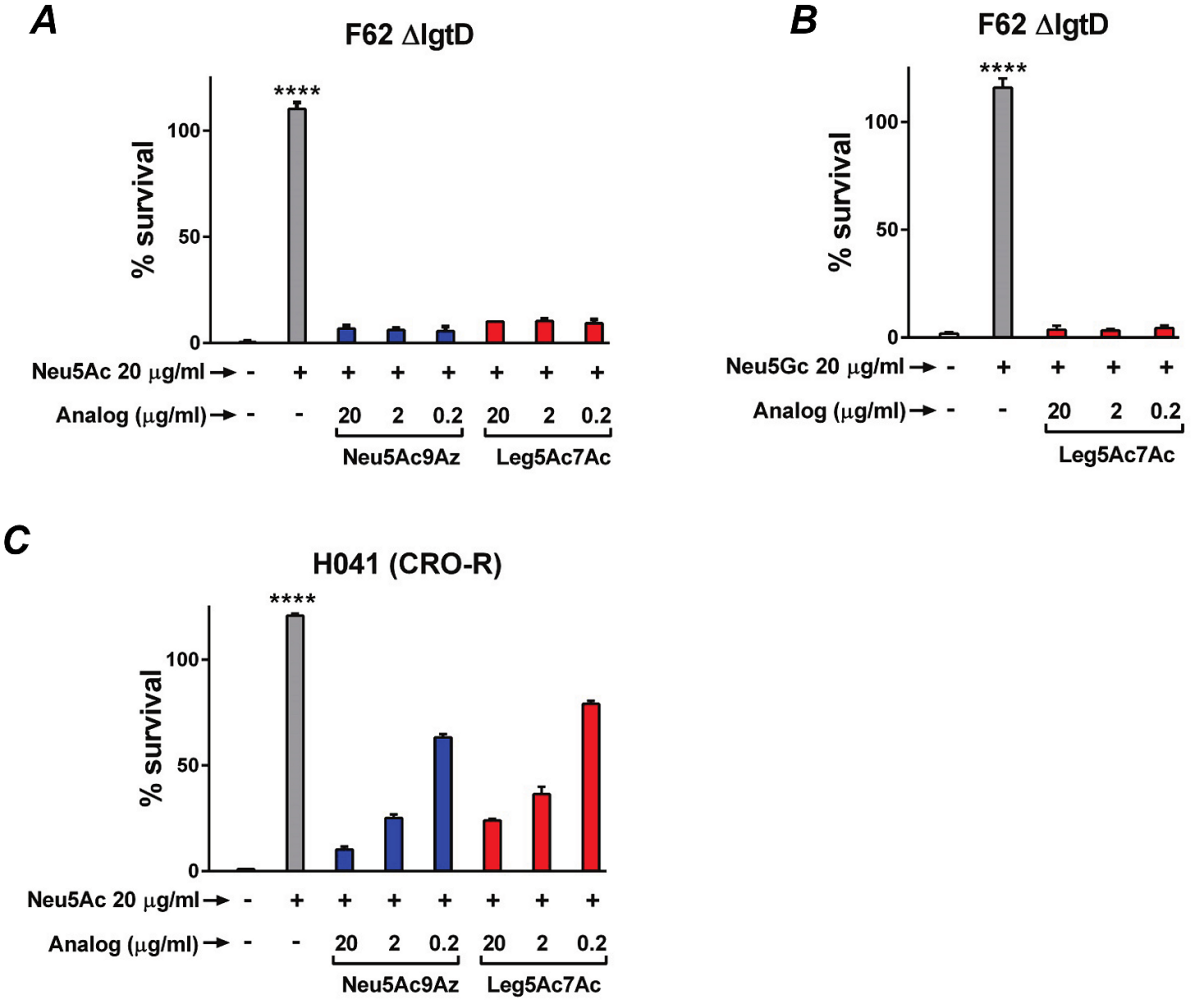
Survival of *N*. *gonorrhoeae* (*Ng)* first incubated in CMP-Neu5Ac or CMP-Neu5Gc followed by incubation with CMP-NulOs. *Ng* F62 ΔlgtD ***(A and B)*** and ceftriaxone-resistant (CRO-R) *Ng* H041 ***(C)*** were suspended in media containing 20 μg/ml CMP-Neu5Ac or CMP-Neu5Gc as indicated for 15 min at 37°C. Following incubation, CMP-Neu5Ac9Az or CMP-Leg5Ac7Ac (at concentrations of 20, 2 or 0.2 μg/ml) were added to bacterial suspensions and then incubated for 2 h. Bacteria were serially diluted and serum bactericidal assays were performed using 2000 CFU and 10% normal human serum (NHS). Controls included bacteria in media that lacked any CMP-Sia altogether or bacteria that were incubated in CMP-Neu5Ac or CMP-Neu5Gc only. Y-axis, % survival (mean (SD) of two independent experiments is shown). ****, P<0.0001 compared to all other reactions (ANOVA).

We next asked whether the two NulOs, Neu5Ac9Az and Leg5Ac7Ac, that were incorporated into gonococcal LOS but did not enhance serum resistance, could prevent the ability of CMP-Neu5Ac to enhance serum resistance of *N*. *gonorrhoeae*. These CMP-activated analogs were added at concentrations of 20, 2 or 0.2 μg/ml to growth media 15 min after the addition of CMP-Neu5Ac (20 μg/ml) to *Ng* F62 ΔlgtD (Fig 6A), and bacteria were allowed to grow for 2 h, followed by bactericidal assays using 10% NHS. As shown in Fig 6A, both CMP-Neu5Ac9Az and CMP-Leg5Ac7Ac prevented serum resistance mediated by CMP-Neu5Ac, even when the latter was present in a 100-fold molar excess. Adding CMP-Leg5Ac7Ac and CMP-Neu5Ac to the growth media simultaneously, each a concentration of 20 μg/ml, also yielded similar results—the mean survival of *Ng* F62 ΔlgtD was only 7.6%. Similarly, CMP-Leg5Ac7Ac prevented CMP-Neu5Gc-mediated serum resistance (Fig 6B). CMP-Neu5Ac9Az and CMP-Leg5Ac7Ac also prevented Neu5Ac-mediated serum resistance of a ceftriaxone-resistant (CRO-R) isolate, H041 (Fig 6C), however prevention was less than with *Ng* F62 ΔlgtD (minimal prevention seen with CMP-NulO concentrations of 0.2 μg/ml). In the unsialylated state, H041 is intrinsically more serum resistant than F62 ΔlgtD (H041 shows >100% survival in 3.3% NHS, while F62 shows <10% survival in 3.3% NHS).

### Competing NulOs block Neu5Ac-mediated complement inhibition

To define the mechanism whereby Neu5Ac9Az and Leg5Ac7Ac blocked complement resistance by Neu5Ac, we studied Ab binding to, and complement component deposition on *Ng* F62 ΔlgtD that was incubated with CMP-Neu5Ac (20 μg/ml), followed 15 min later with either CMP-Neu5Ac9Az or CMP-Leg5Ac7Ac at concentrations of 20, 2 or 0.2 μg/ml. Controls included bacteria grown in the absence of any Sia, or bacteria grown in CMP-Neu5Ac alone.

**Fig 7:**
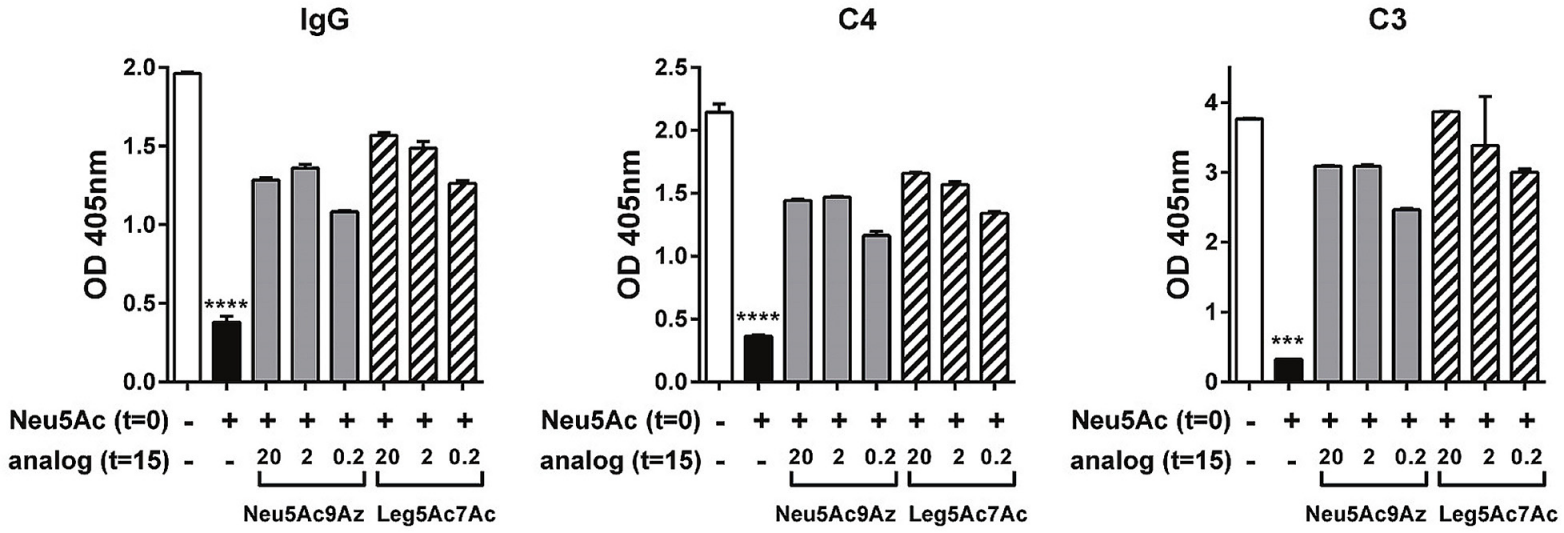
CMP-Neu5Ac9Az and CMP-Leg5Ac7Ac interfere with inhibition of the classical and alternative pathways of complement mediated by CMP-Neu5Ac. *N*. *gonorrhoeae (Ng)* F62 ΔlgtD was incubated with CMP-Neu5Ac for 15 min followed by addition of CMP-Neu5Ac9Az or CMP-Leg5Ac7Ac (at concentrations of 20, 2 or 0.2 μg/ml) for 2 h as described in Fig 6. Bacteria were incubated in 10% normal human serum (NHS) and IgG binding and complement C3 and C4 fragment deposition was measured by ELISA as described in Fig 5. Mean (±SD) of two independent experiments is shown. Controls with heat-inactivated serum were carried out as described in Fig 5. MAb 2-8C-4-1 that detects the Neisserial lipoprotein H.8 was similar across all groups and confirmed similar capture of bacteria in all wells (OD405_nm_ values ranged from 0.964 to 1.039). ***, P<0.001; ****, P<0.0001 compared to other bars in the same graph (ANOVA).

As shown in Fig 7 (and above in Fig 5), incorporation of Neu5Ac into LOS decreased IgG binding and also diminished deposition of complement components C4 and C3. However, addition of CMP-Neu5Ac9Az or CMP-Leg5Ac7Ac at all concentrations tested resulted in lesser reduction in IgG binding in 10% NHS and also in the corresponding deposition of C4 and C3. Similar results were also seen when a lower concentration of serum, 3.3% NHS was used (S4 Fig).

**Fig 8:**
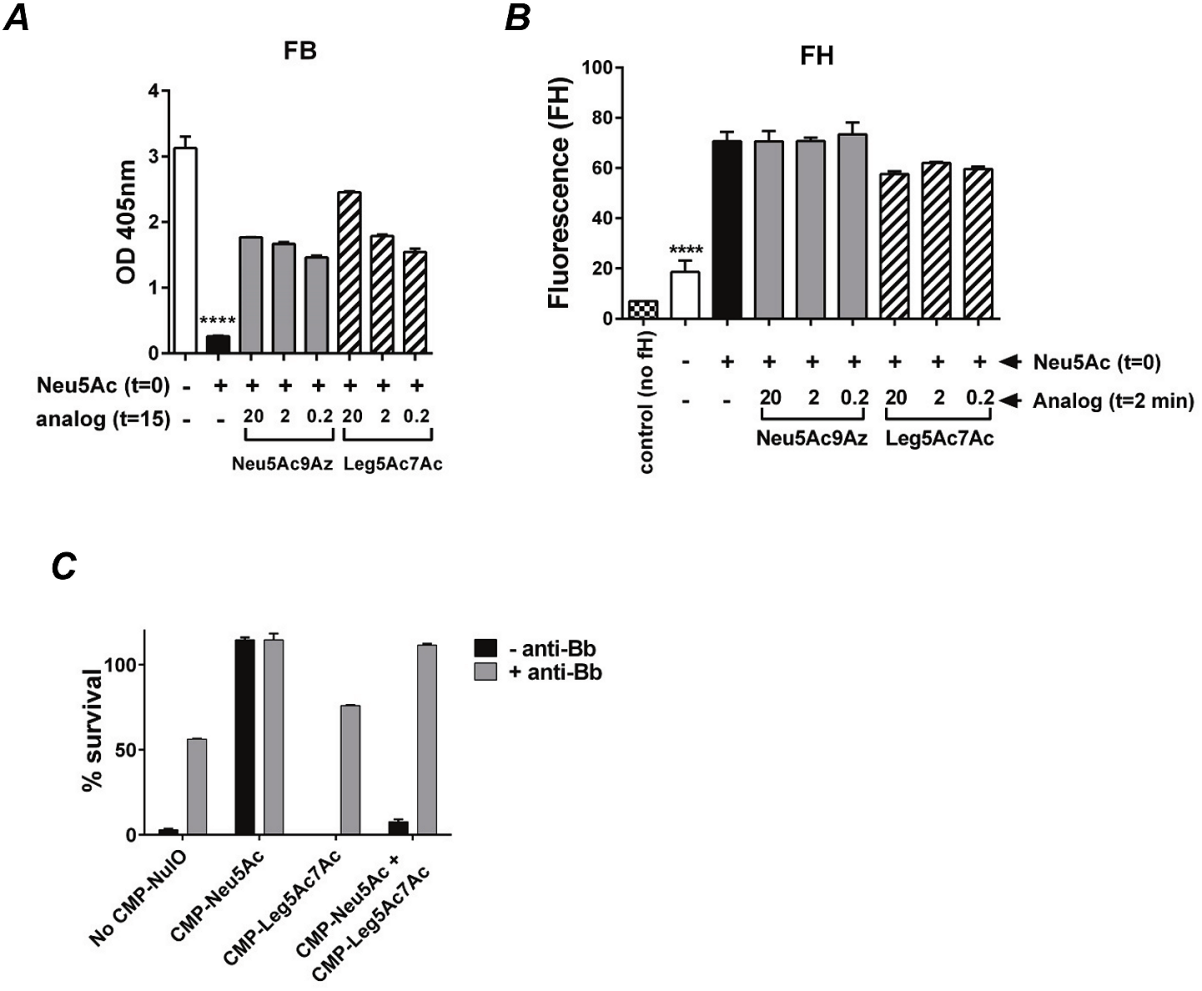
CMP-Neu5Ac9Az and CMP-Leg5Ac7Ac block CMP-Neu5Ac-mediated alternative pathway inhibition despite continued factor H (FH) binding. *N*. *gonorrhoeae (Ng)* F62 ΔlgtD was incubated in media that contained CMP-Neu5Ac (20 μg/ml), followed by addition of either CMP-Neu5Ac9Az or CMP-Leg5Ac7Ac (20, 2 or 0.2 μg/ml). Bacteria were grown for 2 h and factor B (FB) binding (***A***) to bacteria following incubation with 10% normal human serum (NHS) was measured by whole cell ELISA as described in Fig 7. Binding to purified FH (***B***) was measured by flow cytometry using polyclonal goat anti-human FH. The Y-axis represents OD_405nm_ for FB binding and median fluorescence of FH binding. Mean (SD) of two independent observations is shown for each measurement. ****, P<0.0001 (ANOVA) compared to other reactions. ***C***. Alternative pathway activation is required for maximal killing of *Ng* F62 ΔlgtD grown in CMP-Leg5Ac7Ac-containing media. Bacteria were grown in media alone, or media containing CMP-Neu5Ac (20 μg/ml) or CMP-Leg5Ac7Ac (20 μg/ml) or both CMP-NulOs together (each at 20 μg/ml) as indicated on the X-axis. Serum bactericidal assays were performed using normal human serum (all complement pathways intact; black bars) or NHS treated with an anti-factor Bb mAb that blocks FB function (AP inactivated, but classical and lectin pathways intact; grey bars). Y-axis, percent survival. The mean (range) of two independent experiments is shown.

CMP-Neu5Ac9Az and CMP-Leg5Ac7Ac also resulted in lesser reduction in FB deposition (Figs 8A and S4); surprisingly these NulOs did not interfere with FH binding to *Ng* F62 ΔlgtD (Fig 8B). These data seem paradoxical because under physiological conditions, FH functions to irreversibly dissociate factor Bb from C3bBb and also prevents factor B from binding C3; thus, FH and FB/FBb binding usually bear inverse proportions (reviewed in [13]). The relatively unimpeded activation of the alternative pathway, evidenced by high FB deposition seen in the presence of the competing CMP-NulOs that occurs in the face of FH binding, may suggest impaired FH function when Leg5Ac7Ac or Neu5Ac9Az are expressed on the bacterial surface concomitantly with Neu5Ac.

Ab-dependent classical pathway activation is essential for killing of *N*. *gonorrhoeae* [14,15]; disabling the classical pathway abrogates killing of otherwise fully serum sensitive gonococci [15]. To define the role of the alternative pathway in mediating killing by CMP-Leg5Ac7Ac, the function of FB was blocked with an anti-factor Bb mAb [16]. Blocking the alternative pathway enabled unsialylated *Ng* F62 ΔlgtD to survive 56% in the pooled NHS used in these assays (Fig 8C). As expected growth of bacteria in CMP-Neu5Ac abrogated killing (CFU at t_30_ was 115% of the CFUs at t_0_) (Fig 8C). Addition of CMP-Leg5Ac7Ac (20 μg/ml) alone to media, or 15 min after the addition of CMP-Neu5Ac (20 μg/ml), resulted in 76% and 112% survival, respectively, when FB function was blocked (Fig 8C). By contrast (and consistent with the data in Fig 6A), the parallel control bactericidal reactions with all pathways intact (i.e. no anti-Bb) showed 0% survival when bacteria were grown in CMP-Leg5Ac7Ac alone, increasing only to 8% when this NulO was added to media 15 min after CMP-Neu5Ac. Thus, interfering with the function of LOS Neu5Ac by CMP-Leg5Ac7Ac on *Ng* F62 ΔlgtD appears to require a functioning alternative pathway.

### *N*. *gonorrhoeae* LOS does not preferentially incorporate Leg5Ac7Ac

A possible explanation for the ‘dominant-suppressive’ effect of Leg5Ac7Ac and Neu5Ac9Az over Neu5Ac in the bactericidal assays in Fig 6 is preferential incorporation of these competing NulOs into gonococcal LOS. However, characterization of Lst enzyme function has revealed that conversion efficiency for *N*. *meningitidis* and *N*. *gonorrhoeae* F62 α2,3-sialyltransferases with CMP-Leg5Ac7Ac as a donor is comparable to that with CMP-Neu5Ac [17]. Further, binding of mAb 3F11 (binds only to unsialylated LNnT; extensions beyond the terminal Gal of LNnT abrogates 3F11 binding) to *Ng* F62 ΔlgtD grown in concentrations ranging from 0.125 to 25 μg/ml of CMP-Neu5Ac or CMP-Leg5Ac7Ac were similar, providing additional evidence that Leg5Ac7Ac (when used alone) is not preferentially added to the *Ng* LNnT LOS (S5 Fig). Finally, the ratio of Leg5Ac7Ac:Neu5Ac incorporated into *Ng* F62 ΔlgtD LOS,after CMP-Leg5Ac7Ac was added to growth media 15 min after CMP-Neu5Ac had been added, was approximately 1:2 (S6 Fig). Collectively, these data provide strong evidence that the effects of Leg5Ac7Ac in preventing the complement-inhibiting effects of Neu5Ac were not because of the preferential addition of Leg5Ac7Ac on to LOS.

### CMP-Leg5Ac7Ac attenuates gonococcal infection in mice

**Fig 9:**
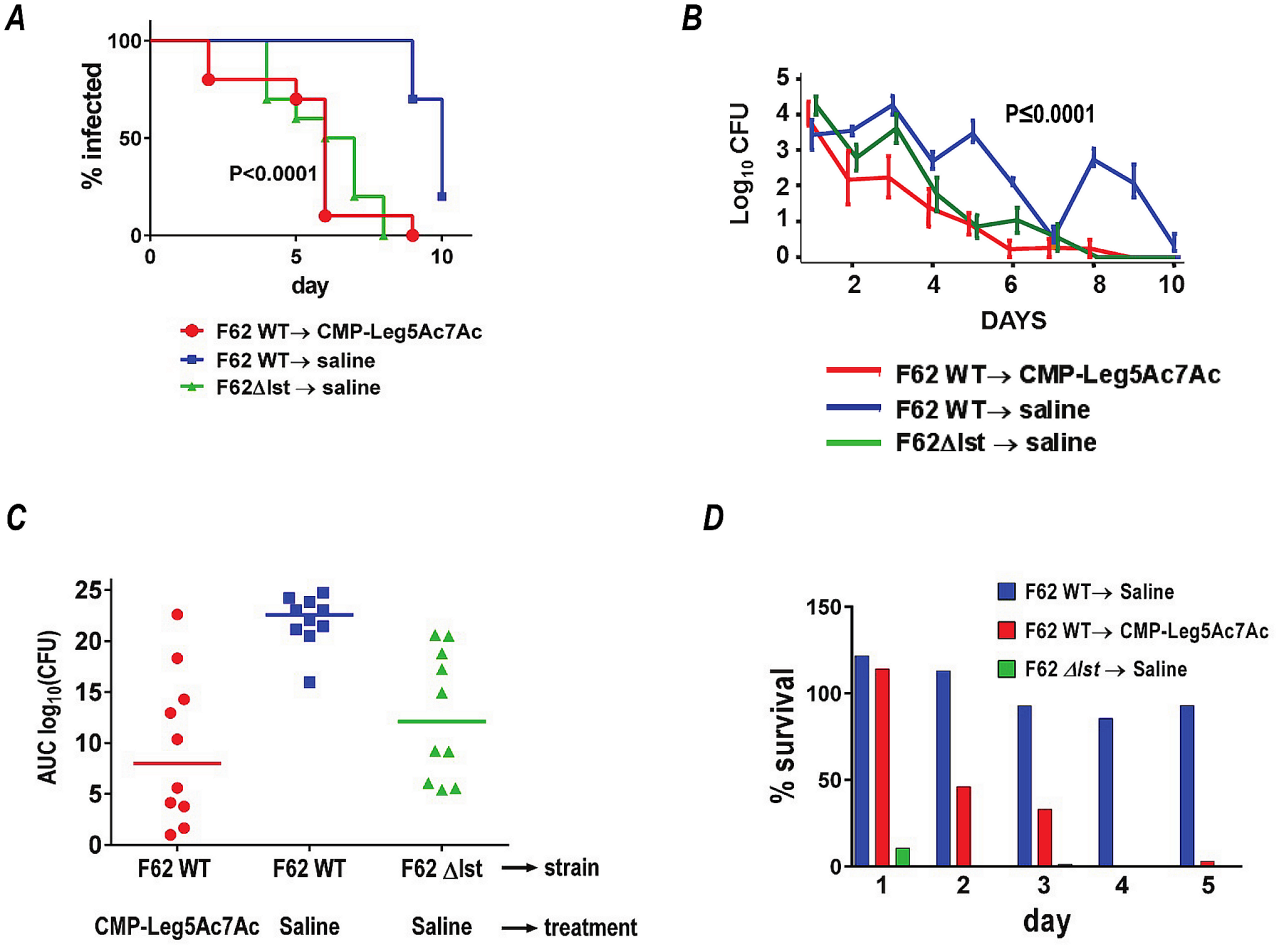
CMP-Leg5Ac7Ac treatment reduces the duration of infection and the burden of *N*. *gonorrhoeae (Ng)* F62 in the BALB/c mouse vaginal colonization model. Three groups of 17β-estradiol treated BALB/c mice (n = 10/group) were inoculated and treated as follows: i) wild-type F62; saline (vehicle control), ii) wild-type *Ng* F62; treated with CMP-Leg5Ac7Ac 10 μg/ml intravaginally daily and iii) *Ng* F62 Δlst; saline. Vaginal swabs were obtained daily to quantify *Ng* CFUs. ***A***. Kaplan Meier analysis of time to clearance. ***B***. Colonization of bacteria (log_10_ CFU) measured daily. ***C***. Bacterial burdens consolidated over time (Area Under the Curve [log _10_ CFU] analysis) for the three groups. There were no statistically significant differences noted between the group treated with CMP-Leg5Ac7Ac and the *Ng* F62 Δlst group. ***D***. Complement resistance of *Ng* recovered directly from the genital tract of mice simulates findings *in vitro*. Equal volumes of vaginal secretions from all mice within a group on each day were pooled, serially diluted and incubated with 10% NHS. Survival of bacteria at 30 min relative to 0 min was measured and the percentage survival expressed on the Y-axis.

Given the urgent need for novel therapies against multidrug-resistant gonorrhea that has spread globally, we next tested the ability of CMP-Leg5Ac7Ac to attenuate gonococcal infection in mice. CMP-Leg5Ac7Ac was chosen because it most effectively blocked Neu5Ac-mediated complement inhibition (Figs 5 and 7 and 8) and because Leg5Ac7Ac-α2,3-lactose-FCHASE substrates are more resistant to microbial and human sialidases (that *Ng* may encounter in the genital tract) than Neu5Ac-α2,3-lactose-FCHASE substrates [18]. Two groups of 17β-estradiol-treated BALB/c mice (10 mice per group) were infected with wild-type strain F62. We used wild-type *Ng* for the experiments *in vivo* because phase variation of LOS (i.e., ‘GalNAc capping’ via LgtD) could constitute a possible mechanism of ‘drug resistance’. One group was treated with 10 μg CMP-Leg5Ac7Ac intravaginally daily; the control group was untreated. A third group of mice (n = 10) was infected with F62 Δ*lst* (unable to sialylate LOS) and served as a control. As shown in Fig 9A, mice treated with CMP-Leg5Ac7Ac cleared infection faster than untreated mice (median clearance time was 6 days in treated mice compared to 10 days for untreated mice; P<0.0001, Kaplan-Meier survival curve; clearance times were compared between groups using a log-rank test). Mixed model analysis indicated significant differences in colonization trends of F62 wild-type over time between the two groups comparing CMP-Leg5Ac7Ac versus untreated mice (P = 0.0005) (Fig 9B). A significant difference in the Mean Areas Under the Curve (mean AUCs) (log_10_ CFU versus time) between the treated and untreated groups challenged with F62 WT was also observed (Fig 9C). To address the possibility that non-specific stimulation of the immune system by CMP-Leg5Ac7Ac may have accounted for its activity, we examined the effects of CMP-Leg5Ac7Ac treatment on the burden of infection caused by an Lst deletion mutant of F62 (F62 *Δlst)* that is unable to add NulO to its LOS. As shown in S7 Fig, CMP-Leg5Ac7Ac treatment did not affect the course of infection (measured as time to clearance or AUC) of F62 *Δlst*.

To confirm that CMP-Leg5Ac7Ac treatment reduced serum resistance of *Ng in vivo*, we incubated bacteria recovered directly from mice (without sub-passage on media) with normal human serum and measured their survival after 30 min. As shown in Fig 9D, the wild-type bacteria from untreated mice remained resistant to complement over all 5 days; bacteria recovered from the CMP-Leg5Ac7Ac treatment group became progressively more serum sensitive over time. As expected, the *Δlst* mutant was uniformly serum sensitive on all days. This experiment was limited to 5 days, because the numbers of bacteria recovered from the treatment and *Δlst* groups thereafter were too low to perform the assay.

**Fig 10:**
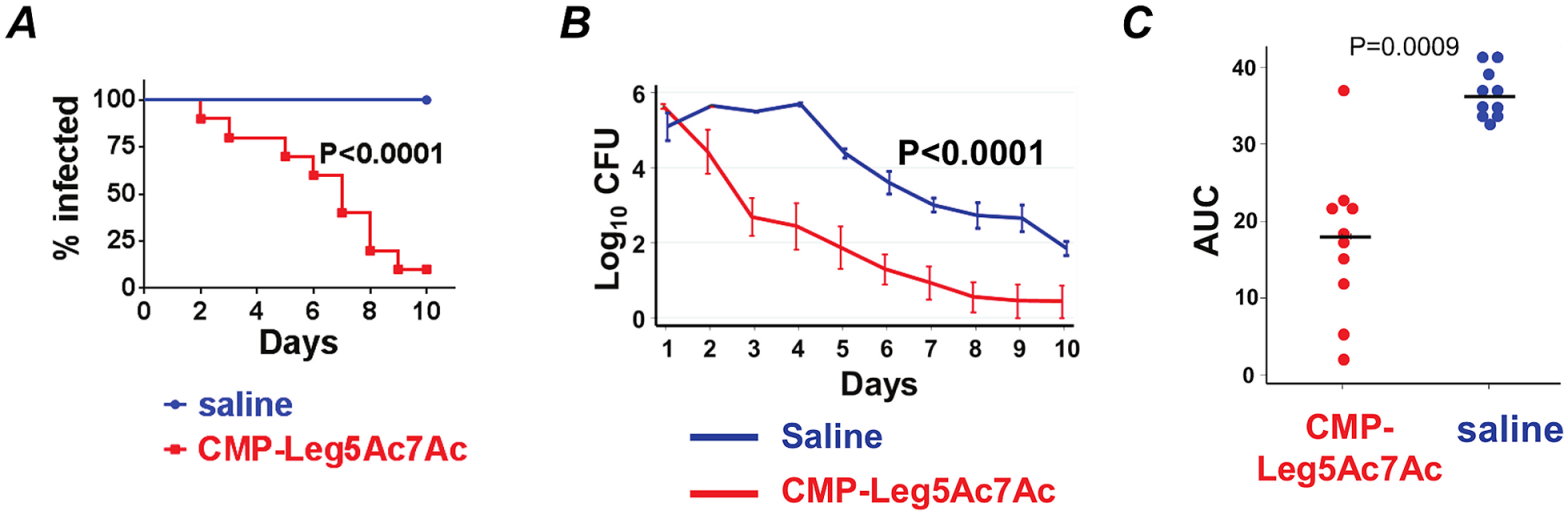
CMP-Leg5Ac7Ac treatment reduces the duration and burden of ceftriaxone-resistant (CRO-R) *N*. *gonorrhoeae (Ng)* H041 in the BALB/c mouse vaginal colonization model. Two groups of 17β-estradiol treated BALB/c mice (n = 10/group) were challenged with 9 x 10^5^ CFU *Ng* H041. One group received CMP-Leg5Ac7Ac 10 μg/ml intravaginally daily; the other group received saline (vehicle control). Vaginal swabs were obtained daily to quantify *Ng* CFUs. ***A***. Kaplan Meier analysis of time to clearance. ***B***. Colonization of bacteria (log_10_ CFU) measured daily. ***C***. Bacterial burdens consolidated over time (Area Under the Curve [log _10_ CFU] analysis) for the two groups.

Similar to findings with F62, CMP-Leg5Ac7Ac significantly attenuated the duration and burden of infection caused by CRO-R isolate H041 in mice (Fig 10). Of note, the H041 isolate is naturally more serum resistant than F62, resulting in minimal clearance at day 10 of saline-treated mice (Fig 10A and 10B).

## Discussion

Over 40 years ago, Ward and colleagues showed that gonococci recovered directly from male urethral secretions fully resisted killing by human complement [19]. However, sub-passage of gonococci on routine culture media abrogated the property of serum resistance and thus was termed ‘unstable’ serum resistance. Almost two decades later, seminal work performed by Smith and co-workers [11,20,21] identified CMP-Neu5Ac as the molecule scavenged by *Ng in vivo* that was responsible for conferring unstable serum resistance. The only molecule modified by Neu5Ac on *Ng* is LOS [9]. Sialylation of LOS is mediated by Lst, an enzyme located on the *Ng* outer membrane [22]. The two gonococcal HepI LOS extensions that can be modified by Neu5Ac are lacto-N-neotetraose (LNnT) (Neu5Acα2–3Galβ1–4GlcNAcβ1–3Galβ1–4Glcβ1–4HepI) and the P^K^ LOS (Neu5Acα2–6Galα1–4Galβ1–4Glcβ1–4HepI) [23]. Here we show that substrate specificity of gonococcal Lst in the context of intact bacteria extends to five additional CMP-Sia analogs listed in Table 1.

Several previous studies have elucidated mechanisms of gonococcal LNnT LOS Neu5Ac-mediated serum resistance and point to regulation of both the classical and alternative pathways of complement [7,8,24,25]. The presence of LOS Neu5Ac on gonococci decreases binding of select mAbs directed against gonococcal PorB [7]. Another report suggested that LOS Neu5Ac did not affect the quantity of anti-LOS IgM binding to bacteria, but perhaps masked epitopes usually recognized by pentameric IgM molecules that resulted in limited engagement of C1q and subsequent activation of the classical pathway [25]. The addition of Neu5Ac via an α2–3 linkage to the terminal Gal of LNnT also enhances binding of human FH, the principal inhibitor of the alternative pathway of complement. Enhancement of FH binding to sialylated gonococci also requires the presence of gonococcal PorB specifically; specifically replacement of gonococcal PorB with meningococcal PorB3 does not augment FH binding [26]. Neu5Ac that enhances FH binding and results in high-level serum resistance of gonococci is also specific for the LNnT LOS structure. Previously we reported that addition of α2–6-linked Neu5Ac to the terminal Gal of the P^K^-like (or L1) LOS did not enhance FH binding and conferred resistance only to lower concentrations of serum relative to serum resistance seen with Neu5Ac α2–3-linked to LNnT LOS [23]. Binding of FH hastens dissociation of alternative pathway C3/C5 convertases (C3b,Bb and C3bC3b,Bb, respectively) [13] and likely contributes to the barely detectable levels of FB on the Neu5Ac and Neu5Gc-bearing strains even in 10% serum.

In contrast to the high-level serum resistance seen with LOS bearing Neu5Ac and Neu5Gc, NulOs with further modifications at C7, C8 and C9 were equally well incorporated and yet conferred only low level serum resistance (Neu5Ac9Ac or Neu5Gc8Me) or no serum resistance (Leg5Ac7Ac and Neu5Ac9Az). These data also suggest that the negative charge of Sias alone is not sufficient for FH binding or complement evasion—Neu5Gc8Me, Leg5Ac7Ac and Neu5Ac9Az are all negatively charged, yet do not enhance FH binding or classical pathway inhibition. In contrast, N-glycolyl substitution at the 5-carbon position of Neu5Ac (to Neu5Gc) facilitates FH binding and increases serum resistance. The importance of the C9 substitutions reported here are in accordance with several previous studies as discussed below. Removal of the 9-carbon was associated with loss of 90% of the complement inhibition by Sia [27]. The extent of 9-O-acetylation of Sias on erythrocytes varies widely across mouse strains and correlates directly with the susceptibility of erythrocytes to lysis by the alternative pathway [28]. Similarly, increased expression of 9-O-acetylated sialoglycans on the red cells of individuals with visceral leishmaniasis is associated with greater alternative pathway activation [29]. Fearon showed that removal of C8 and C9 substitutions of Sia by NaIO_4_ treatment rendered sheep erythrocytes susceptible to lysis by the alternative pathway [3]. Using ligand-based nuclear magnetic resonance (NMR), Blaum et al recently showed that the O8 and O9 hydroxyl groups of the glycerol chain of Neu5Ac that was α2–3-linked to lactose formed hydrogen bonds with the amide and carbonyl groups, respectively, of the W1198 residue in FH domain 20 [5]. In addition the O9 also formed a hydrogen bond with the W1183 backbone [5]. Effective and unimpeded alternative pathway inhibition when the 5Ac group is replaced with 5Gc is also consistent with a prior study [27].

Previously we reported that the addition of Neu5Ac to gonococcal LOS diminished classical pathway activation (less C4 fragment deposition) [12]. In the present study, using relatively low numbers of bacteria in Ab binding and complement deposition assays to more closely simulate conditions in serum bactericidal assays, we observed a substantial reduction in binding of IgG to sialylated (Neu5Ac and Neu5Gc) bacteria. Reduced IgG binding could account, at least in part, for classical pathway inhibition mediated by Neu5Ac. Downstream events, including C4, C3 and FBb deposition (the latter a measure of alternative pathway recruitment), were effectively regulated only in low serum concentrations when LOS expressed Neu5Gc8Me and Neu5Ac9Ac, but were not likely to reach a threshold that permitted bacterial survival in 10% complement. Of note, the intermediate phenotype exhibited by CMP-Neu5Ac9Ac treated bacteria, compared to CMP-Neu5Ac9Az treatment, is likely due to the instability of the C9 O-acetyl group where some of the Neu5Ac9Ac is converted to Neu5Ac, as this instability was observed during preparation and storage of CMP-Neu5Ac9Ac.

Sialic acids (Sias) are present on all vertebrate cell types and play diverse roles in immune function [2]. Humans (and birds and reptiles) lack a functional CMP-Neu5Ac hydroxylase enzyme (CMAH) and therefore cannot convert Neu5Ac to Neu5Gc [30]. In contrast to humans, most other mammals studied (with the exception of ferrets [31] and new world monkeys [32]) including mice, possess a functional CMAH and therefore express both Neu5Ac and Neu5Gc glycoconjugates. Thus, the LOS of *Ng* recovered from mice during experimental studies of gonococcal colonization may be modified with either Neu5Ac and/or Neu5Gc. The ability of Neu5Gc on *Ng* LOS to also inhibit complement validates the use of the mouse model to study complement interactions with gonococci and evaluate vaccines and therapeutics that may rely on complement activation for their efficacy. The observation that Neu5Gc also facilitates binding of human FH and results in high-level serum resistance that simulates the findings with Neu5Ac would validate the use of human FH transgenic mice to elucidate the role of complement in pathogenesis of sialylated gonococci.

Binding of purified FH (independent of C3 fragment deposition as occurs in context of normal serum) to sialylated gonococci is human specific, with weak binding observed to chimpanzee FH [33]. This raises the question of the utility of wild-type mice, whose endogenous FH is not known to bind directly to sialylated gonococci, for studies related to sialic acid. Distinct from the mechanism that involves direct FH binding described above, gonococcal LOS Neu5Ac also regulates non-human (e.g., rhesus macaque) C3 deposition on gonococci, albeit to a lesser extent than seen in species (chimpanzee and human) whose FH directly bind (i.e., in a C3 independent manner) to sialylated bacteria [33]. Although not formally demonstrated on sialylated gonococci, we have shown that Neu5Ac substitution of meningococcal LNnT LOS limits non-human (in this instance, rat) C3 deposition [34]. This mechanism represents a cooperative interaction of the C-terminal FH domains with C3 fragments that bind to bacteria and Neu5Ac-LNnT [34] that is concordant with the prior model proposed by Kajander and colleagues [4]. A key role for sialic acid in bacterial survival *in vivo* in wild-type mice has been shown in Fig 10 (lower ‘virulence’ of the Lst mutant), and replicates prior findings [35,36].

We and others previously have proposed a role for bacterial Neu5Ac (reviewed in [6]), including Leg5Ac7Ac in immune evasion [37,38], possibly through increased FH binding. However, in the context of *Ng*, the current data (Figs 4B and S3) refute this notion for Leg5Ac7Ac. Paradoxically, incorporation of Leg5Ac7Ac by *Ng* LOS appears to confer heightened susceptibility of *Ng* to the immune system.

*Ng* has developed widespread resistance to almost every conventional antibiotic currently in clinical use [39]. High-level resistance to ceftriaxone was first reported in 2011 [40,41] and has ushered in an era of potentially untreatable gonorrhea. There is an urgent need for novel therapeutics and vaccines against this disease [42,43]. LOS sialylation is an important component of gonococcal pathogenesis; isogenic mutants that cannot sialylate their LOS are at a disadvantage *in vivo* compared to their wild-type counterparts [44]. Manipulation of gonococcal LOS ‘sialylation’ could represent a novel preventive or treatment strategy. We acknowledge that further work is needed to elucidate the mechanistic details of the ‘dominant negative’ effect of Leg5Ac7Ac/ Neu5Ac9Az over Neu5Ac substitution of gonococcal LOS, however given the dire need for alternative therapeutics against drug-resistant gonorrhea, we decided to focus first on the practical utility of these findings. Here we have shown that select derivatives of Sia, such as Neu5Ac9Az and Leg5Ac7Ac block Neu5Ac-mediated serum resistance (Fig 6). *In vitro*, the effects of these CMP-Sia derivatives are also evident when added to bacteria shortly after adding CMP-Neu5Ac. *In vivo*, however, bacteria actively replicate for several generations and are likely to incorporate the competing Sia analog over time. Of note, the effects of CMP-Neu5Ac were negated even when CMP-Neu5Ac9Az or CMP-Leg5Ac7Ac were provided at ~100-fold lower concentrations. These competing CMP-NulOs diminished inhibition of the classical pathway (determined by measuring both increased IgG binding and C4 deposition) and serum resistance elicited by CMP-Neu5Ac. Surprisingly, FBb continued to bind to *Ng* in an uninhibited manner (Fig 8A) in the face of high FH binding (Fig 8B). This raises the possibility that FH is rendered ‘non-functional’ (i.e., does not display decay accelerating activity and liberate FBb from C3/C5 convertases) when Leg5Ac7Ac- or Neu5Ac9Az-substituted LOS and Neu5Ac-subsituted LOS are concomitantly expressed on the bacterial surface. The reasons for the ‘dominant-suppressive’ effect of the competing NulOs over complement inhibition by Neu5Ac remain to be elucidated. Based on the phylogeny of NulOs [45] it is likely that *Ng* Lst may have evolved from an ancestral transferase that originally used CMP-bacterial NulOs such as CMP- Leg5Ac7Ac as substrates.

In addition to its ability to counteract Neu5Ac-mediated complement inhibition effectively, CMP-Leg5Ac7Ac was chosen to study therapeutic efficacy *in vivo* because of the sialidase resistance exhibited by Leg5Ac7Ac, as evidenced by the resistance of Leg5Ac7Ac-α(2,3)-lactose-FCHASE to mammalian and microbial sialidases [18]. Sialidases are present in the female genital tract [46,47,48,49,50]; resistance of Leg5Ac7Ac-substituted LNnT on *Ng* to sialidases would be a desired attribute for a therapeutic CMP-NulO. Indeed, mice treated with CMP-Leg5Ac7Ac cleared wild-type *Ng* F62 more rapidly (Fig 9A) and harbored lower bacterial burdens over the course of infection (Fig 9B and 9C) analogous to clearance and colonization levels using a Lst knockout mutant of strain F62. Furthermore, as daily treatment of mice with CMP-Leg5Ac7Ac took hold, surviving bacteria isolated from mice during the course of infection became progressively more sensitive to killing by complement (Fig 9D), consistent with organisms having incorporated Leg5Ac7Ac (Figs 2 and 3). Under the influence of CMP-Leg5Ac7Ac, conversion of the infecting strain into a phenotype unable to sialylate only with Neu5Ac (and/or Neu5Gc in the mouse [51]) resulted in an attenuation of bacterial burden similar to that seen with the Lst mutant, which manifests incomplete virulence in the mouse model of gonococcal infection [36]. In contrast to strain F62, gonococcal strain H041 is fully resistant to ceftriaxone (CRO-R), resistant to 3.3% serum, and more resistant to the bactericidal properties conferred by NuIOs Neu5Ac9Az and Leg5Ac7Ac than strain F62 Δ*lgtD* (Fig 6A and 6C). Nevertheless, mice treated with CMP-Leg5Ac7Ac also cleared strain H041 more rapidly and harbored lower bacterial burdens than saline-treated infected animals (Fig 10).

In conclusion, these findings have shed new light on the molecular basis of gonococcal LOS sialylation and how this translates to complement regulation on the bacterial surface. CMP-Sia analogs that interfere with serum resistance of gonococci mediated by CMP-Neu5Ac may predict their efficacy as topical anti-gonococcal therapeutics in vivo. The identification of such Sia analogs that can interfere with a key virulence mechanism of *Ng* paves the way to develop novel therapeutic strategies against rapidly spreading multi-drug resistant gonococcal infections.

## Materials and Methods

### Synthesis of CMP-Nonulosonate (CMP-NulO) compounds

Neu5Ac, Neu5Gc and Neu5Ac9Az nonulosonates were purchased from commercial sources (Inalco Pharmaceuticals, Sigma, Sussex Research Laboratories Inc.). Neu5Ac9Ac and Neu5Gc8Me were synthesized using published methods [52,53]. Leg5Ac7Ac and Pse5Ac7Ac were prepared enzymatically using methods in [38] and [37], respectively. CMP-activation of nonulosonate sugars was performed enzymatically using appropriate CMP-Sia, CMP-legionaminic acid and CMP-pseudaminic acid synthetases from either *N*. *meningitidis*, *C*. *jejuni* or *Helicobacter pylori* [37,38,54,55,56]. Reactions contained 50 mM Tris pH 8–9, 50 mM MgCl_2_, 15.7 mM CTP, 15 mM nonulosonate, 4 units pyrophosphatase per mM of CTP, and sufficient quantities of CMP-NulO synthetase enzyme to obtain optimal conversion at 5–6 hours. CMP-NulO enzymatic reaction mixtures were then passed through an Amicon Ultra-15 (10,000 molecular weight cut-off) or Ultra-4 (5,000 molecular weight cut-off) filter membrane before purification. Filtered CMP-NulO samples were then lyophilized and desalted/purified using a Superdex Peptide 10/300 GL (GE Healthcare) column in ammonium bicarbonate or NaCl solutions. To achieve additional purity, elution fractions containing individual CMP-NulOs were subjected to anion-exchange chromatography (Mono Q 4.6/100 PE, GE Healthcare) using either an ammonium bicarbonate or NaCl gradient. When NaCl gradients were used, CMP-NulOs were ‘desalted’ by gel filtration (Superdex Peptide 10/300 GL) using 1 mM NaCl. Quantification of CMP-NulO preparations was determined using the molar extinction coefficient of CMP (ε260 = 7,400). Prior to lyophilization, CMP-NuIOs were suspended in sodium hydroxide or NaCl solutions at molar ratios of 1:2 (NuIO: salt).

Purified CMP-nonulosonates were dissolved in >99% D_2_O and structural analysis performed by mass spectroscopy (MS) using either a Varian Inova 500 MHz (^1^H) spectrometer with a Varian Z-gradient 3 mm probe or a Varian 600 MHz (^1^H) spectrometer with a Varian 5 mm Z-gradient probe. All spectra were referenced to an internal acetone standard (δ_H_ 2.225 ppm and δ_C_ 31.07 ppm). Results that are shown in S1 Table and S1 Fig verify the production and purity of each CMP-nonulosonate compound made for this study; CMP-LegAc7Ac and CMP-Pse5Ac7Ac, which were confirmed based on NMR data presented in [38] and [37].

CMP-NulOs were also characterized using capillary electrophoresis (CE)-MS analysis. Separation of ions was achieved by CE (Prince CE system [Prince Technologies, Netherlands]) in a 90 cm long bare fused-silica capillary (365 μm OD x 50 μm ID). The 30 mM morpholine aqueous running CE buffer (adjusted to pH9 with formic acid) was coupled with the capillary sheath fluid (isopropanol: methanol [2:1]) at their interface prior to mass spectrometry (API3000 mass spectrometer [Applied Biosystems/Sciex, Concord, ON, Canada]). S2 Table indicates measured m/z ions of each CMP-nonulosonate compound using CE-MS. Measurements correspond to calculated masses.

### Bacterial strains and growth conditions

A mutant of *Ng* strain F62 [57] that lacked expression of lipooligosaccharide glycosyltransferase D *(lgtD)*, called F62 ΔlgtD [58], was provided by Dr. Daniel C. Stein (University of Maryland). LgtD adds a GalNAc residue to the terminal Gal of the HepI lacto-N-neotetraose species [10]. Therefore, any extension of the HepI of F62 ΔlgtD is limited to the addition of a NulO residue that is transferred from the CMP-NulO added to growth media.

Bacteria (F62 ΔlgtD) grown overnight on chocolate agar plates were suspended in gonococcal liquid media supplemented with IsoVitaleX [59] that contained specified concentrations of the CMP-NulO. Bacteria were then incubated at 37°C for the period specified in each experiment.

A spontaneous streptomycin-resistant mutant of *Ng* F62 [60], kindly provided by Dr. Ann E. Jerse, was used in mouse infection studies. An isogenic *lst* deletion mutant, derived from this strain, was constructed using plasmid pUC18-lst-Kan [23].

Strain H041 (sequence type (ST) 7363; NG-MAST ST 4220) was isolated from a female commercial sex-worker in Kyoto, Japan [40]. This isolate is highly resistant to ceftriaxone (MIC 2–4 μg/ml) and to several other antibiotics [40]. H041 was rendered resistant to streptomycin by transformation with *rpsL* derived from streptomycin resistant *Ng* strain FA1090. Both wildtype H041 and streptomycin resistant H041 expressed predominantly the sialylatable HepI lacto-*N*-neotetraose LOS, confirmed by silver stain and western blot with mAb 3F11.

### Antibodies

Anti-FH mAb (Quidel, catalog no. A254 (mAb 90X)) or goat anti-human FH were used in flow cytometry assays to detect human FH binding to bacteria. Goat polyclonal antibodies against C3, C4 and factor B (FB) were obtained from Complement Technology, Inc (Tyler, TX). Anti-factor Bb (Quidel) added to serum at 100 μg/ml was used to block factor B function [16], thereby disabling the alternative pathway in serum bactericidal assays. Alkaline phosphatase conjugated anti-human IgG and IgM were purchased from Sigma (St. Louis, MO). mAb 3F11 (mouse IgM, kindly provided by Dr. Michael A. Apicella, University of Iowa) binds to the unsialylated HepI lacto-*N*-neotetraose structure; sialylation of LOS results in decreased binding of mAb 3F11 [61]. MAb 2-8C-4-1 [62] recognizes Neisserial H.8 lipoprotein and was used in whole cell ELISA assays to measure capture of bacteria on microtiter wells. FITC conjugated anti-mouse IgG and anti-goat IgG, and alkaline phosphatase conjugated anti-mouse IgM, anti-mouse IgG and anti-goat IgG were all obtained from Sigma. Neu5Gc incorporation into LNnT LOS was detected using a Neu5Gc-specific chicken polyclonal IgY Ab (1:2,000) [63] followed by FITC conjugated donkey anti-chicken IgY secondary Ab (1:200; Jackson ImmunoResearch).

### SDS-PAGE and Western blotting for LOS analysis

Gonococcal lysates treated with protease K (100 μg/ml) and NuPAGE LDS Sample Buffer (4X) (Invitrogen) were separated on NuPAGE 12% Bis-Tris (Invitrogen) gels using Novex MES running buffer (Invitrogen) followed by transfer to an Immobilon PVDF membrane (Millipore) by western blotting. Membranes were blocked with PBS/1% milk and probed with tissue culture supernatants that contained mAb 3F11. mAb 3F11-reactive LOS bands were disclosed with anti-mouse IgM conjugated to alkaline phosphatase followed by the addition of BCIP/NBT-Purple Liquid Substrate (Sigma). Silver staining for LOS was performed following electrophoresis on 16.5% Criterion Tris-Tricine gels (Bio-Rad) using the Bio-Rad Silver Stain kit.

### Mass spectrometry of gonococcal LOS

*Ng* F62 ΔlgtD was grown in media containing the CMP salts of Neu5Ac, Neu5Gc, Neu5Ac9Ac, Neu5Gc8Me, Neu5Ac9Az and Leg5Ac7Ac and LOS was extracted, de-O-acylated and analyzed by MS as described previously [64].

### Flow cytometry for factor H binding

Factor H (FH) binding to bacteria was performed using flow cytometry as described previously [16]. Briefly, bacteria (*Ng* F62 ΔlgtD) were harvested from chocolate agar plates and grown in liquid media that contained the specified concentration of the CMP-NulO as described above. Bacteria were then washed with Hanks Balanced Salt Solution (HBSS) containing 1mM Ca^2+^ and 1 mM Mg^2+^ (HBSS^++^) and incubated with FH purified from human plasma (Complement Technology, Inc.; concentration specified for each experiment). Bound FH was detected using either anti-FH mAb (Quidel, catalog no. A254 (mAb 90X)) or affinity-isolated polyclonal goat anti-human FH, followed by FITC conjugated anti-mouse IgG or anti-goat IgG, respectively (Sigma); both Abs had similar performance characteristics. All reaction mixtures were carried out in HBSS^++^/1% BSA in a final volume of 50 μl. Flow cytometry was performed using a FACSCalibur instrument (Becton Dickinson) and data were analyzed using FlowJo (version 7.2.5; Tree Star, Inc.).

### Whole cell ELISA for complement component deposition

C3 and C4 fragment deposition on, and FB binding to bacteria were measured by whole cell ELISA as described previously [12,16]. Briefly, ~10^6^ organisms in HBSS^++^ were incubated with NHS (at concentrations of 3.3% or 10%) in a reaction volume of 100 μl for 10 min at 37°C. This time point was chosen based on the kinetics of complement deposition on gonococci in previously published data [12]. Reactions were stopped after 10 min by washing three times with ice-cold HBSS containing 5 mM phenylmethylsulfonyl fluoride at 4°C. Organisms were resuspended in 200 μl of the same buffer, and 50 μl of each sample applied per well of a 96-well U-bottomed polystyrene microtiter plate (Dynatech Laboratories, Inc., Chantilly, VA) and incubated for 3 h at 37°C. Plates were washed with PBS containing 0.05% Tween 20. Primary antibodies (polyclonal goat anti-human C3, C4 and FB) were diluted in PBS, and secondary antibodies diluted in PBS-0.05% Tween 20 prior to use. To ensure similar capture of bacteria incubated under different conditions, we measured the amount of gonococcal H.8 antigen [65] expression using mAb 2-8C-4-1 [62], followed by anti-mouse IgG-alkaline phosphatase conjugate.

### Serum bactericidal assay

Serum bactericidal assays were performed as described previously [59]. Bacteria harvested from an overnight culture on chocolate agar plates and ~10^5^ CFU of *Ng* were grown in liquid media containing the specified concentration of CMP-Sia as specified for each experiment. Bacteria were diluted in Morse A and ~2000 CFU of *Ng* F62 ΔlgtD were incubated with NHS (concentration specified for each experiment). The final reaction volumes were maintained at 150 μl. Aliquots of 25 μl of reaction mixtures were plated onto chocolate agar in duplicate at the beginning of the assay (t_0_) and again after incubation at 37°C for 30 min (t_30_). Survival was calculated as the number of viable colonies at t_30_ relative to t_0_.

To measure the sensitivity of bacteria recovered directly from mouse secretions to human complement, an equal aliquot of secretions from each mouse within each group were pooled (pools were made to ensure adequate number of bacteria for the assay over the first 5 days). Secretions from each pool were serially diluted, each dilution was incubated with 10% NHS and survival at t_30_ relative to t_0_ was carried out as described above. Only those dilutions that yielded ~100–300 CFU at t_0_ were considered in order to ensure similar bacteria-to-complement ratio across groups.

### Mouse vaginal colonization model

Female BALB/c mice 5–6 weeks of age (Jackson Laboratories) in the diestrus phase of the estrous cycle were started on treatment (that day) with 0.5 mg of water soluble 17β-estradiol (Sigma) in 200 μl of water given subcutaneously on each of three days; −2, 0 and +2 days (before, the day of and after inoculation) to prolong the estrus phase of the cycle and promote susceptibility to *Ng* infection. Antibiotics (vancomycin, colistin, neomycin, trimethoprim and streptomycin) ineffective against *Ng* were also used to reduce competitive microflora [66]. Mice (n = 20) were then infected with 3.4 x 10^5^ CFU of strain F62. One group of mice (n = 10) was treated with 10 μg CMP-Leg5Ac7Ac (1 mg/ml in sterile H_2_O) daily intravaginally while the remaining 10 mice (controls) were given saline (vehicle control). A third group (n = 10) was infected with 2.5 x 10^5^ CFU of *Ng* F62 Δlst (unable to sialylate) and received no treatment. In a separate control experiment, *Ng* F62 Δlst infection was compared with *Ng* F62Δ*lst* infection ‘treated’ with CMP-Leg5Ac7Ac.

### Statistics

Experiments that compared clearance of *N*. *gonorrhoeae* in independent groups of mice estimated and tested three characteristics of the data [67]: Time to clearance, longitudinal trends in mean log_10_ CFU and the cumulative CFU as area under the curve (AUC). Statistical analyses were performed using mice that initially yielded bacterial colonies on Days 1 and/or 2. Median time to clearance was estimated using Kaplan-Meier survival curves; the times to clearance were compared between groups using a log-rank test. Mean log_10_ CFU trends over time were compared between groups using a linear mixed model with mouse as the random effect using both a random intercept and a random slope. A quadratic function in time was determined to provide the best fit; random slopes were also quadratic in time. A likelihood ratio test was used to compare nested models (with and without the interaction term of group and time) to test whether the trend differed over time between the two groups. The mean AUC (log_10_CFU vs. time) was computed for each mouse to estimate the bacterial burden over time (cumulative infection); the means under the curves were compared between groups using the nonparametric rank sum test because distributions were skewed or kurtotic.

### Ethics statement

Collection of human sera and its use were approved by the University of Massachusetts Medical School Institutional Review Board (IRB). Informed, written consent was obtained from all serum donors (Docket # H00005614). Use of animals in this study was performed in strict accordance with the recommendations in the Guide for the Care and Use of Laboratory Animals of the National Institutes of Health. The protocol was approved by the Institutional Animal Care and Use Committee (IACUC) at the University of Massachusetts Medical School (Docket # A-1930).

### Online supplemental material

S1 Fig shows the purity of the CMP salts of Neu5Ac, Neu5Ac9Ac, Neu5Ac9Az, Neu5Gc and Neu5Gc8Me. S2 Fig is a schematic representation of the complement cascade. S3 Fig shows representative histogram tracings of FH binding to *Ng* F62 ΔlgtD grown in the presence of different CMP-Sia analogs. S4 Fig shows Ig binding to and complement component deposition on *Ng* F62 ΔlgtD following incubation with 3.3% NHS; bacteria were grown in media containing CMP-Neu5Ac alone, or CMP-Neu5Ac plus increasing concentrations of either CMP-Leg5Ac7Ac or CMP-Neu5Ac9Az. S5 Fig shows a comparison of mAb 3F11 binding to *Ng* F62 ΔlgtD grown in increasing concentrations of either CMP-Neu5Ac or CMP-Leg5Ac7Ac. S6 Fig shows the relative amounts of Neu5Ac and Leg5Ac7Ac incorporation by Ng F62 Δ*lgtD* LOS, each strain grown alone. S7 Fig shows the time to clearance (left graph) and Area Under Curve (AUC) of F62 Δlst infection of BALB/c mice that were given saline control or treated with CMP-Leg5Ac7Ac.

S1 Table shows NMR chemical shifts for CMP-NulOs prepared in this study. S2 Table shows CE-MS data of purified CMP-NulO sugars prepared in this study. S3 Table shows mass spectrometric analysis of Ng F62 ΔlgtD LOS sialylated with different NuIOs.

## Supporting Information

S1 Fig The ^1^H spectrum and ^1^H-^13^C HSQC correlation spectrum of different CMP-nonulosonic acids used in this study. Spectra for CMP-Pse5Ac7Ac and CMP-Leg5Ac7Ac have been described previously [37,38]. Spectra were recorded on a Varian Inova Unity 500 MHz spectrometer with standard Varian pulse sequences in D_2_O at 25°C, with 16 scans for the ^1^H spectrum and 64 scans for HSQC. C, cytosine; R, ribose; N, Nonulosonic acid; NAc, 5-NHAc CH3 regions of either Neu5Ac, Neu5Ac9Ac, or Neu5Ac9Az; OAc, 9-OAc CH3 region of Neu5Ac9Ac; Me, 8-OMe region of Neu5Gc8Me; Gc, glycolyl region of Neu5Gc or Neu5Gc8Me. Acetone was included as an internal reference.
(TIFF)

S2 Fig Schematic representing activation/regulation of the complement cascade. The fragments released into solution are indicated in blue font. The key fluid-phase regulators are indicated in green font. KEY: CRP, C-reactive protein; SAP, serum amyloid P component; PTX3, pentraxin 3; C1 inh, C1 inhibitor; α2-M, α2-macroglobulin; C4BP, C4b-binding protein; FHL-1, factor H like protein-1. From *Ram S*, *Lewis LA*, *Rice PA*. *Clin Microbiol Rev*. *2010*. *23(4)*:*740–780*.
(TIFF)

S3 Fig Factor H (FH) binding to *N*. *gonorrhoeae* (*Ng)* grown in media containing CMP-Sia analogs. Representative histogram tracings of an experiment depicted in Fig 3. X-axis, fluorescence (log_10_ scale); Y-axis, counts (linear scale). Numbers alongside each histogram represent the median fluorescence. Control represents bacteria that were incubated in buffer alone (no added FH).
(TIFF)

S4 Fig CMP-Neu5Ac9Az and CMP-Leg5Ac7Ac interfere with inhibition of the classical and alternative pathways of complement mediated by CMP-Neu5Ac. *N*. *gonorrhoeae(Ng)* F62 ΔlgtD was incubated with 20 μg/ml Neu5Ac for 15 min followed by addition of CMP-Neu5Ac9Az or CMP-Leg5Ac7Ac (at concentrations of 20, 2 or 0.2 μg/ml) for 2 h as described in Fig 5. Bacteria were incubated in 3.3% NHS and IgG and IgM binding and deposition of complement components C3, C4 and FB was measured by ELISA. *Ng* H.8 lipoprotein was performed to measure bacterial capture to microtiter wells. Mean (±SD) of two independent experiments is shown.
(TIFF)

S5 Fig Neu5Ac and Leg5Ac7Ac are incorporated into Ng LNnT LOS with similar efficiency. *N*. *gonorrhoeae* (*Ng*) F62 ΔlgtD was grown in media alone, or media containing decreasing concentrations (2-fold dilutions ranging from 25 μg/ml to 0.125 μg/ml) of CMP-Neu5Ac or CMP-Leg5Ac7Ac. Binding of mAb 3F11 was measured by flow cytometry. mAb 3F11 binds only to unsubstituted LNnT LOS; extensions beyond the terminal Gal of LNnT abrogates 3F11 binding. Y-axis, median fluorescence (mean (SD) of duplicate samples of one experiment); X-axis, CMP-NulO concentration.
(TIFF)

S6 Fig Quantification of the relative amounts of Neu5Ac and Leg5Ac7Ac in the LOS of *N*. *gonorrhoeae (Ng)* strain F62 lgtD grown in media containing no CMP-NulO, or in media containing CMP-Neu5Ac alone (20 μg/ml), CMP-Leg5Ac7Ac alone (20 μg/ml), or in media where CMP-Leg5Ac7Ac was added 15 min after CMP-Neu5Ac (both CMP-NulOs at 20 μg/ml). LOS was extracted on a small-scale from a 12 ml culture volume using a modification of the phenol-chloroform method [68]. ***A***. Estimation of the relative amounts of LOS in the samples. Equal volumes of each preparation taken just prior to the final lyophilization step were loaded on a 4–12% Bis-Tris gel (Invitrogen/Life Technologies) with MES running buffer and LOS was revealed by silver staining. The relative intensities of the LOS bands were estimated using ImageJ software (NIH) and are indicated below each lane. ***B***. Estimation of the relative amounts of Neu5Ac and Leg5Ac incorporated by Ng F62 lgtD lacto-*N*-neotetraose LOS. Lyophilized LOS extracts samples were dissolved in H_2_O. Acid hydrolysis of the NulOs was performed with 0.1 M hydrochloric acid at 80°C for 1 h to release them from the underlying LOS backbone Samples were cooled to room temperature, neutralized with NaOH and then derivatized with 1,2-diamino-4,5-methylene-dioxybenzene (DMB) and analyzed by high performance liquid chromatography (HPLC), as described below. The DMB derivatization reagent was made by mixing 14 mM DMB (Sigma), 18 mM sodium hydrosulfite, 0.75 M 2-mercaptoethanol, and 1.6 M acetic acid, followed by incubation at 50°C for 2.5 h [69,70]. DMB-derivatized samples were analyzed on a LaChrom Elite HPLC System (Hitachi) using a Phenomenex Gemini 5μ C18 250 × 4.6-mm HPLC column at room temperature. Fluorescence was detected at 448 nm using excitation at 373 nm. To separate NulOs, an isocratic solvent composition of 88% water, 7% methanol and 5% acetonitrile was used at a flow rate of 0.9 mL/min; the data collection time was expanded to 90 min. A Neu5Ac standard curve is shown on the left. Relative amounts of each of the NulOs (normalized to the respective LOS band densities) are indicated on the graph on the right as the normalized peak area.
(TIFF)

S7 Fig CMP-Leg5Ac7Ac treatment does not alter the course of infection with *Ng* F62 *Δ*lst. BALB/c mice were infected with a LOS sialyltransferase deletion mutant of *Ng* F62 (F62 *Δ*lst) and given either saline (vehicle control; black lines/circles; n = 10 mice) or CMP-Leg5Ac7Ac (red line/squares; n = 10 mice) and bacterial burdens monitored daily. The left graph shows time to clearance of infection and the right graph compares the Areas Under Curves (AUCs) across the two groups.
(TIFF)

S1 Table NMR chemical shifts for CMP-nonulosonates prepared in this study. All spectra were referenced to an internal acetone standard (δ_H_ 2.225 ppm and δ_C_ 31.07 ppm).
(DOC)

S2 Table CE-MS data of purified CMP-nonulosonate sugars prepared in this study. The detected ions indicated here were not present in negative control samples.
(DOCX)

S3 Table MS analysis of LOS of *Ng* F62 lgtD grown in CMP-NulOs. (DOCX)
