# Supplementary figures and images for: Utilizing CMP-Sialic Acid Analogs to Unravel Neisseria gonorrhoeae Lipooligosaccharide-Mediated Complement Resistance and Design Novel Therapeutics
Source: PLoS Pathog. 2015 Dec 2;11(12):e1005290. doi: 10.1371/journal.ppat.1005290 (PMC4668040; doi:10.1371/journal.ppat.1005290)

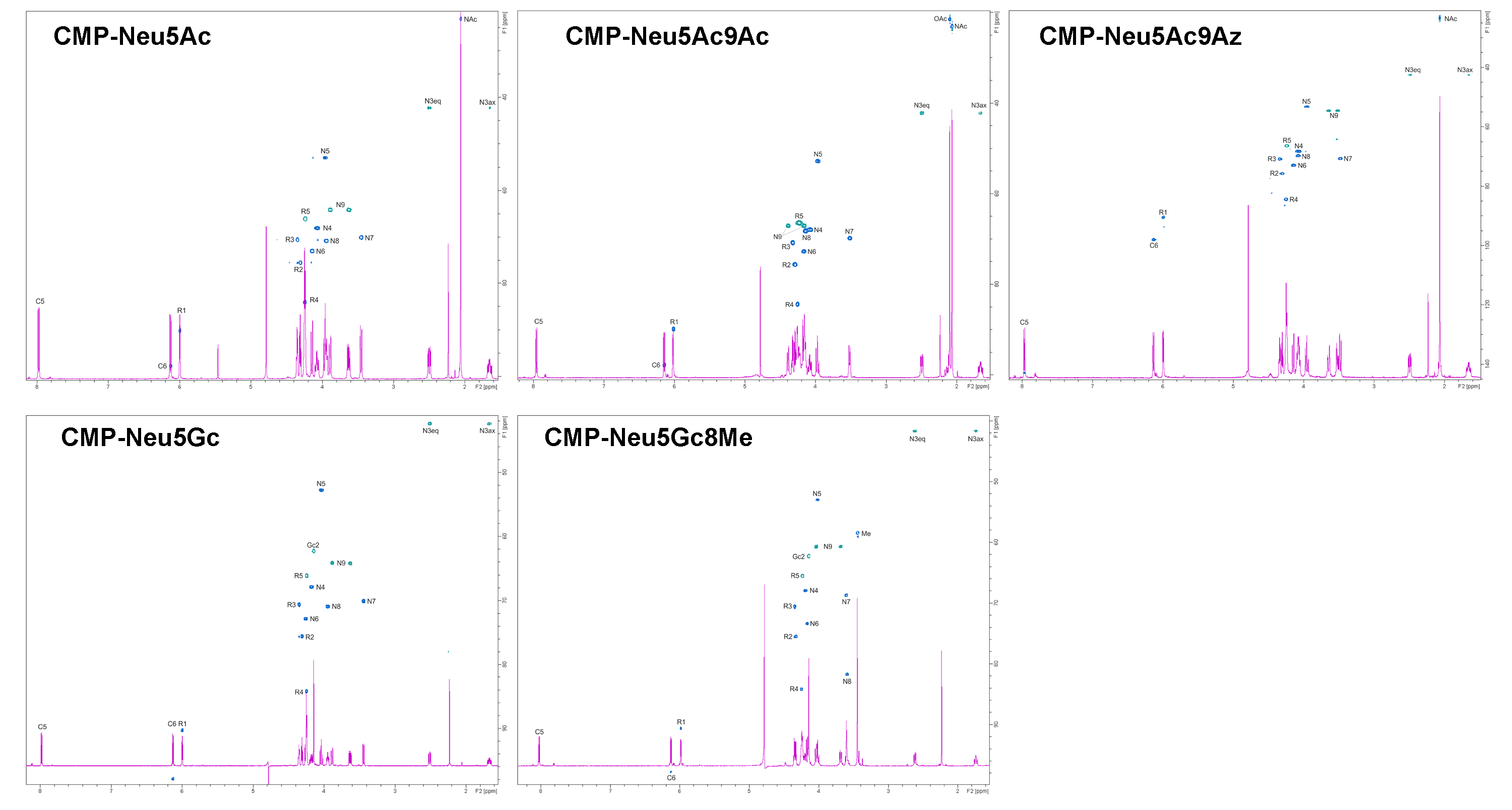

Supplement: S1 Fig — Spectra for CMP-Pse5Ac7Ac and CMP-Leg5Ac7Ac have been described previously [37,38]. Spectra were recorded on a Varian Inova Unity 500 MHz spectrometer with standard Varian pulse sequences in D2O at 25°C, with 16 scans for the 1H spectrum and 64 scans for HSQC. C, cytosine; R, ribose; N, Nonulosonic acid; NAc, 5-NHAc CH3 regions of either Neu5Ac, Neu5Ac9Ac, or Neu5Ac9Az; OAc, 9-OAc CH3 region of Neu5Ac9Ac; Me, 8-OMe region of Neu5Gc8Me; Gc, glycolyl region of Neu5Gc or Neu5Gc8Me. Acetone was included as an internal reference. (TIFF) [file ppat.1005290.s001.tiff]

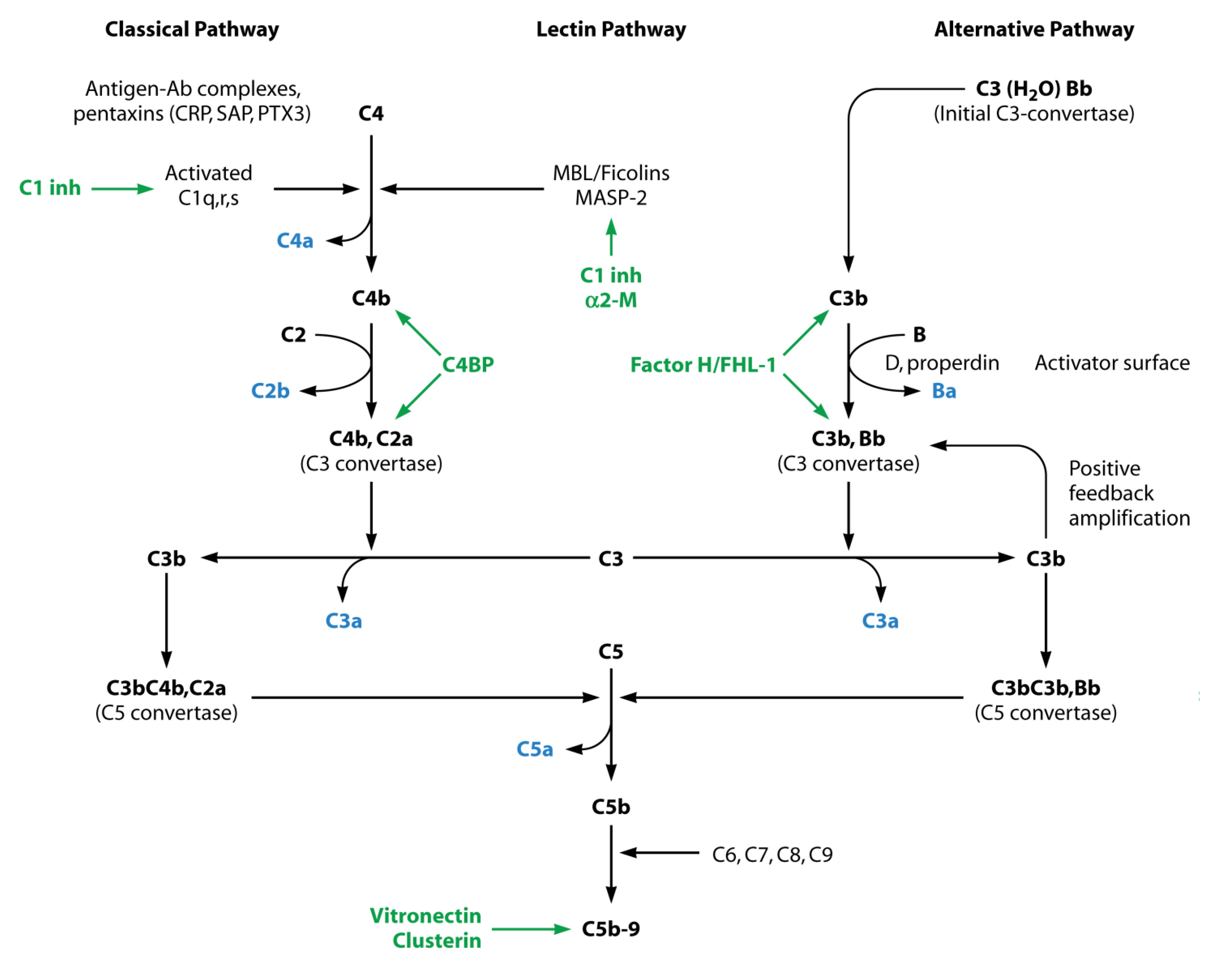

Supplement: S2 Fig — The fragments released into solution are indicated in blue font. The key fluid-phase regulators are indicated in green font. KEY: CRP, C-reactive protein; SAP, serum amyloid P component; PTX3, pentraxin 3; C1 inh, C1 inhibitor; α2-M, α2-macroglobulin; C4BP, C4b-binding protein; FHL-1, factor H like protein-1. From Ram S, Lewis LA, Rice PA. Clin Microbiol Rev. 2010. 23(4):740–780. (TIFF) [file ppat.1005290.s002.tiff]

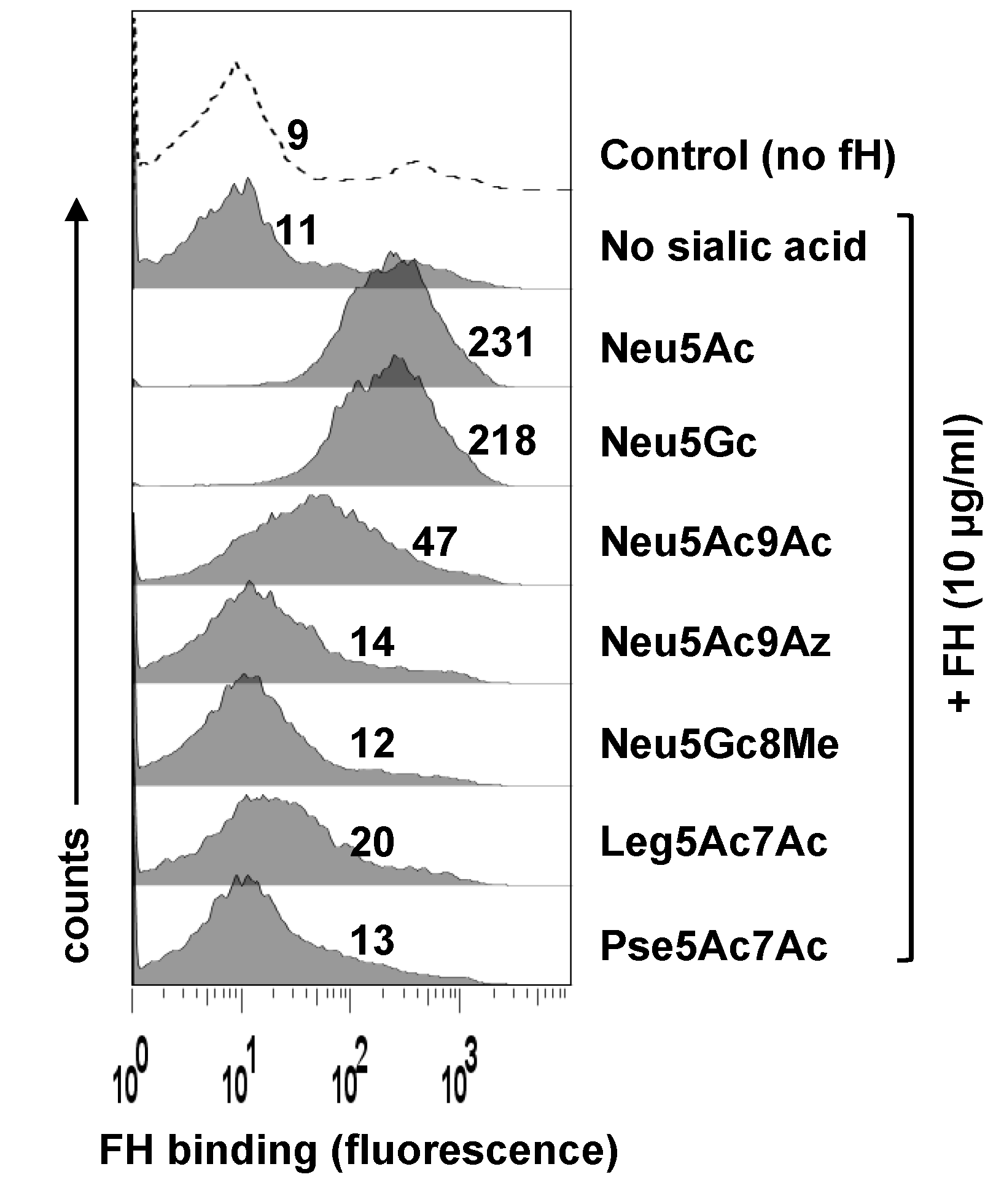

Supplement: S3 Fig — Representative histogram tracings of an experiment depicted in Fig 3. X-axis, fluorescence (log10 scale); Y-axis, counts (linear scale). Numbers alongside each histogram represent the median fluorescence. Control represents bacteria that were incubated in buffer alone (no added FH). (TIFF) [file ppat.1005290.s003.tiff]

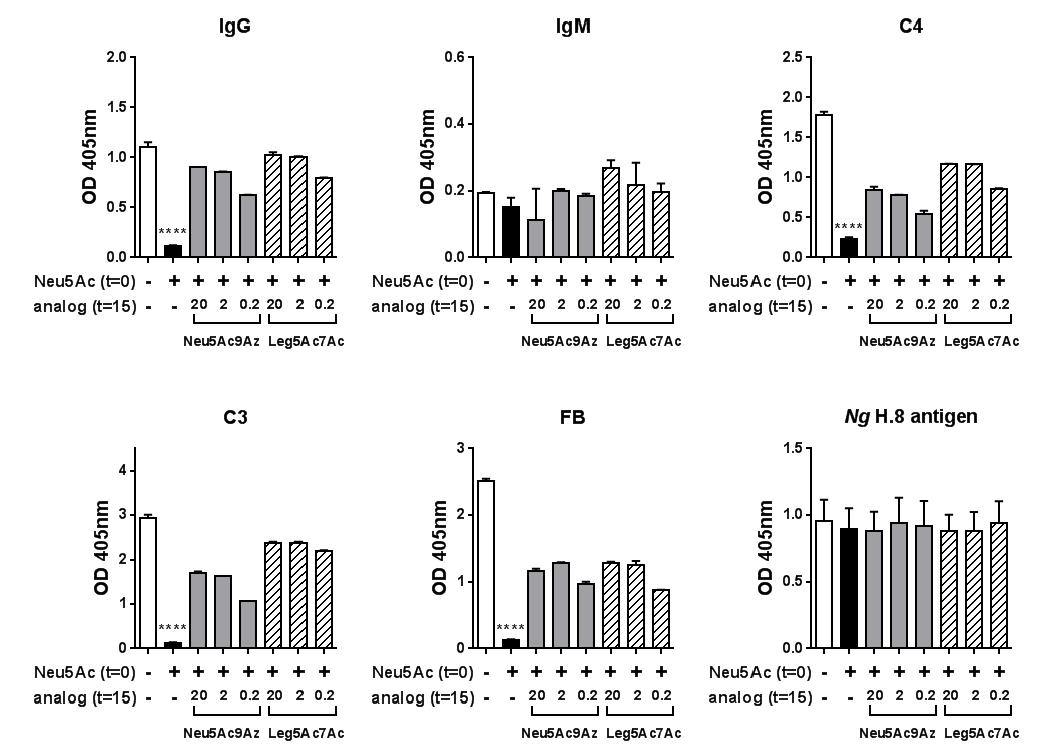

Supplement: S4 Fig — N. gonorrhoeae(Ng) F62 ΔlgtD was incubated with 20 μg/ml Neu5Ac for 15 min followed by addition of CMP-Neu5Ac9Az or CMP-Leg5Ac7Ac (at concentrations of 20, 2 or 0.2 μg/ml) for 2 h as described in Fig 5. Bacteria were incubated in 3.3% NHS and IgG and IgM binding and deposition of complement components C3, C4 and FB was measured by ELISA. Ng H.8 lipoprotein was performed to measure bacterial capture to microtiter wells. Mean (±SD) of two independent experiments is shown. (TIFF) [file ppat.1005290.s004.tiff]

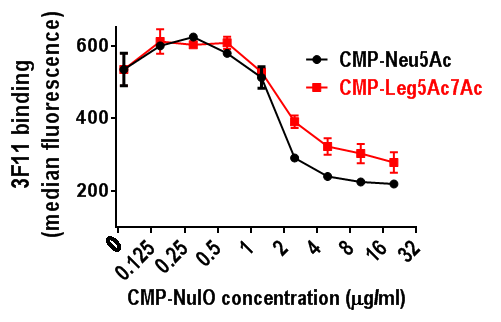

Supplement: S5 Fig — N. gonorrhoeae (Ng) F62 ΔlgtD was grown in media alone, or media containing decreasing concentrations (2-fold dilutions ranging from 25 μg/ml to 0.125 μg/ml) of CMP-Neu5Ac or CMP-Leg5Ac7Ac. Binding of mAb 3F11 was measured by flow cytometry. mAb 3F11 binds only to unsubstituted LNnT LOS; extensions beyond the terminal Gal of LNnT abrogates 3F11 binding. Y-axis, median fluorescence (mean (SD) of duplicate samples of one experiment); X-axis, CMP-NulO concentration. (TIFF) [file ppat.1005290.s005.tiff]

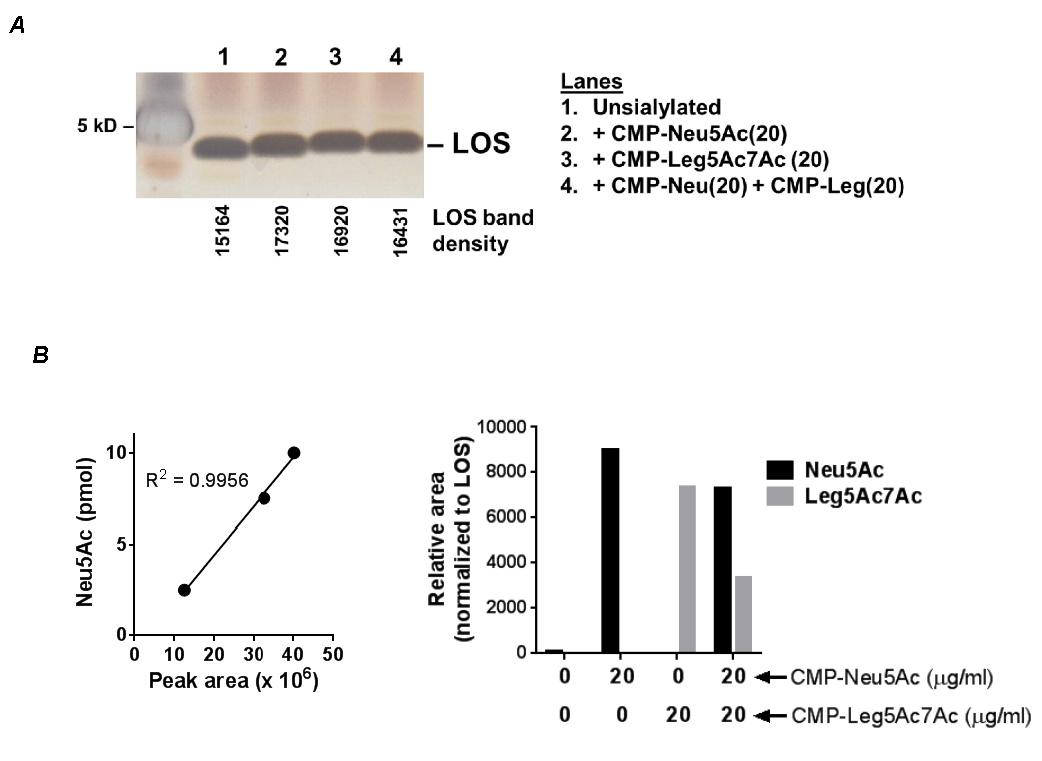

Supplement: S6 Fig — LOS was extracted on a small-scale from a 12 ml culture volume using a modification of the phenol-chloroform method [68]. A. Estimation of the relative amounts of LOS in the samples. Equal volumes of each preparation taken just prior to the final lyophilization step were loaded on a 4–12% Bis-Tris gel (Invitrogen/Life Technologies) with MES running buffer and LOS was revealed by silver staining. The relative intensities of the LOS bands were estimated using ImageJ software (NIH) and are indicated below each lane. B. Estimation of the relative amounts of Neu5Ac and Leg5Ac incorporated by Ng F62 lgtD lacto-N-neotetraose LOS. Lyophilized LOS extracts samples were dissolved in H2O. Acid hydrolysis of the NulOs was performed with 0.1 M hydrochloric acid at 80°C for 1 h to release them from the underlying LOS backbone Samples were cooled to room temperature, neutralized with NaOH and then derivatized with 1,2-diamino-4,5-methylene-dioxybenzene (DMB) and analyzed by high performance liquid chromatography (HPLC), as described below. The DMB derivatization reagent was made by mixing 14 mM DMB (Sigma), 18 mM sodium hydrosulfite, 0.75 M 2-mercaptoethanol, and 1.6 M acetic acid, followed by incubation at 50°C for 2.5 h [69,70]. DMB-derivatized samples were analyzed on a LaChrom Elite HPLC System (Hitachi) using a Phenomenex Gemini 5μ C18 250 × 4.6-mm HPLC column at room temperature. Fluorescence was detected at 448 nm using excitation at 373 nm. To separate NulOs, an isocratic solvent composition of 88% water, 7% methanol and 5% acetonitrile was used at a flow rate of 0.9 mL/min; the data collection time was expanded to 90 min. A Neu5Ac standard curve is shown on the left. Relative amounts of each of the NulOs (normalized to the respective LOS band densities) are indicated on the graph on the right as the normalized peak area. (TIFF) [file ppat.1005290.s006.tiff]

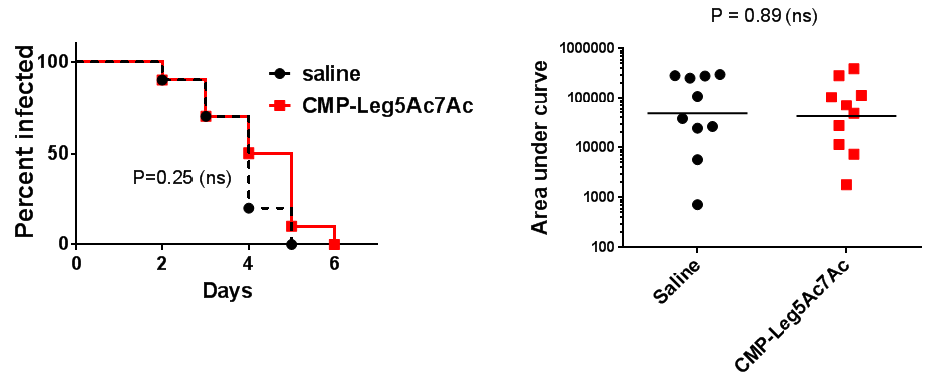

Supplement: S7 Fig — BALB/c mice were infected with a LOS sialyltransferase deletion mutant of Ng F62 (F62 Δlst) and given either saline (vehicle control; black lines/circles; n = 10 mice) or CMP-Leg5Ac7Ac (red line/squares; n = 10 mice) and bacterial burdens monitored daily. The left graph shows time to clearance of infection and the right graph compares the Areas Under Curves (AUCs) across the two groups. (TIFF) [file ppat.1005290.s007.tiff]
