# Supplementary material for: Utilizing CMP-Sialic Acid Analogs to Unravel Neisseria gonorrhoeae Lipooligosaccharide-Mediated Complement Resistance and Design Novel Therapeutics
Source: PLoS Pathog. 2015 Dec 2;11(12):e1005290. doi: 10.1371/journal.ppat.1005290 (PMC4668040; doi:10.1371/journal.ppat.1005290)
Supplement: S1 Table — All spectra were referenced to an internal acetone standard (δH 2.225 ppm and δC 31.07 ppm). (DOC) [file ppat.1005290.s008.doc]

**S1 Table.** NMR chemical shifts for CMP-nonulosonates prepared in this study. All spectra were referenced to an internal acetone standard (δ_H_ 2.225 ppm and δ_C_ 31.07 ppm)

NMR chemical shifts δ (ppm) for CMP-5-acetamido-3,5-dideoxy-D-*glycero*-D-*galacto*-nonulosonic acid (CMP-Neu5Ac).

|  | H3ax | 1.65 |  |  |
| --- | --- | --- | --- | --- |
|  | H3eq | 2.49 | C3 | 42.2 |
|  | H4 | 4.07 | C4 | 68.0 |
|  | H5 | 3.95 | C5 | 52.8 |
|  | H6 | 4.14 | C6 | 72.9 |
|  | H7 | 3.45 | C7 | 70.0 |
|  | H8 | 3.94 | C8 | 70.7 |
|  | H9 | 3.62; 3.89 | C9 | 64.0 |

NMR chemical shifts δ (ppm) for CMP-5-hydroxyacetamido-3,5-dideoxy-D-*glycero*-D-*galacto*-nonulosonic acid (CMP-Neu5Gc).

|  | H3ax | 1.67 |  |  |
| --- | --- | --- | --- | --- |
|  | H3eq | 2.51 | C3 | 42.4 |
|  | H4 | 4.18 | C4 | 67.8 |
|  | H5 | 4.04 | C5 | 52.7 |
|  | H6 | 4.25 | C6 | 72.8 |
|  | H7 | 3.44 | C7 | 70.0 |
|  | H8 | 3.95 | C8 | 70.9 |
|  | H9 | 3.63; 3.88 | C9 | 64.2 |

|  |  |  |  |  |
| --- | --- | --- | --- | --- |
|  |  |  |  |  |
|  |  |  |  |  |
|  |  |  |  |  |
|  |  |  |  |  |
|  |  |  |  |  |
|  |  |  |  |  |
|  |  |  |  |  |

NMR chemical shifts δ (ppm) for CMP-5-acetamido-9-azido-3,5,9-trideoxy-D-*glycero*-D-*galacto*-nonulosonic acid (CMP-Neu5Ac9Az).

|  | H3ax | 1.65 |  |  |
| --- | --- | --- | --- | --- |
|  | H3eq | 2.49 | C3 | 42.4 |
|  | H4 | 4.07 | C4 | 68.2 |
|  | H5 | 3.95 | C5 | 53.1 |
|  | H6 | 4.15 | C6 | 72.9 |
|  | H7 | 3.48 | C7 | 70.5 |
|  | H8 | 4.07 | C8 | 69.7 |
|  | H9 | 3.51; 3.64 | C9 | 54.5 |

NMR chemical shifts δ (ppm) for CMP-5-acetamido-9-O-acetyl-3,5-dideoxy-D-*glycero*-D-*galacto*-nonulosonic acid (CMP-Neu5Ac9Ac).

|  | H3ax | 1.66 |  |  |
| --- | --- | --- | --- | --- |
|  | H3eq | 2.49 | C3 | 42.1 |
|  | H4 | 4.08 | C4 | 67.9 |
|  | H5 | 3.97 | C5 | 52.8 |
|  | H6 | 4.17 | C6 | 72.7 |
|  | H7 | 3.51 | C7 | 69.7 |
|  | H8 | 4.15 | C8 | 68.1 |
|  | H9 | 4.16; 4.39 | C9 | 67.1 |

|  |  |  |  |  |
| --- | --- | --- | --- | --- |
|  |  |  |  |  |
|  |  |  |  |  |
|  |  |  |  |  |
|  |  |  |  |  |
|  |  |  |  |  |
|  |  |  |  |  |
|  |  |  |  |  |

NMR chemical shifts δ (ppm) for CMP-5-hydroxyacetamido-8-O-methyl-3,5-dideoxy-D-*glycero*-D-*galacto*-nonulosonic acid (CMP-Neu5Gc8Me).

|  | H3ax | 1.74 |  |  |
| --- | --- | --- | --- | --- |
|  | H3eq | 2.62 | C3 | 41.6 |
|  | H4 | 4.20 | C4 | 67.9 |
|  | H5 | 4.01 | C5 | 52.9 |
|  | H6 | 4.16 | C6 | 73.3 |
|  | H7 | 3.60 | C7 | 68.7 |
|  | H8 | 3.59 | C8 | 81.7 |
|  | H9 | 3.68; 4.03 | C9 | 60.7 |
