# Supplementary material for: Utilizing CMP-Sialic Acid Analogs to Unravel Neisseria gonorrhoeae Lipooligosaccharide-Mediated Complement Resistance and Design Novel Therapeutics
Source: PLoS Pathog. 2015 Dec 2;11(12):e1005290. doi: 10.1371/journal.ppat.1005290 (PMC4668040; doi:10.1371/journal.ppat.1005290)
Supplement: S2 Table — The detected ions indicated here were not present in negative control samples. (DOCX) [file ppat.1005290.s009.docx]

**S2 Table.** CE-MS data of purified CMP-nonulosonate sugars prepared in this study. The detected ions indicated here were not present in negative control samples.

| **Compound** | **Observed**  **m/z** | **Calculated**  **mass** | **Formula**  **(M)** | **Comments** |
| --- | --- | --- | --- | --- |
| CMP-Pse5Ac7Ac | 638.5 | 639.5 | C22H34O15N5P | [M-H]^-^ |
| CMP-Leg5Ac7Ac | 638.4 | 639.5 | C22H34O15N5P | [M-H]^-^ |
| CMP-Neu5Ac | 613.5 | 614.4 | C20H31N4O16P | [M-H]^-^ |
| CMP-Neu5Gc | 629.3 | 630.5 | C20H31N4O17P | [M-H]^-^ |
| CMP-Neu5Gc8Me | 643.3 | 644.5 | C21H33N4O17P | [M-H]^-^ |
| CMP-Neu5Ac9Ac | 655.5 | 656.5 | C22H33N4O17P | [M-H]^-^ |
| CMP-Neu5Ac9Az | 638.3 | 639.5 | C20H30N7O15P | [M-H]^-^ |
