# Supplementary material for: Utilizing CMP-Sialic Acid Analogs to Unravel Neisseria gonorrhoeae Lipooligosaccharide-Mediated Complement Resistance and Design Novel Therapeutics
Source: PLoS Pathog. 2015 Dec 2;11(12):e1005290. doi: 10.1371/journal.ppat.1005290 (PMC4668040; doi:10.1371/journal.ppat.1005290)
Supplement: S3 Table — (DOCX) [file ppat.1005290.s010.docx]

**S3 Table. MS analysis of LOS of *Ng* F62 lgtD grown in CMP-NulOs**

| **CMP-NulO added** | **Detected ions** | **Det. Mass (Da)** | **Theoretical mass (Da)** | **Compositions** |
| --- | --- | --- | --- | --- |
| CMP-Neu5Ac | 1027.5 | 3085.5 | 3084.8 | 1Neu5Ac, 3Hex, 2HexNAc, 2Hep, 2Kdo, 1PE, LipidA-OH |
|  | 1068.5 | 3208.5 | 3207.9 | 1Neu5Ac, 3Hex, 2HexNAc, 2Hep, 2Kdo, 2PE, LipidA-OH |
|  | 1076 | 3231 | 3229.9 | 1Neu5Ac, 3Hex, 2HexNAc, 2Hep, 2Kdo, 2PE, LipidA-OH + Na^A^ |
|  |  |  |  |  |
| CMP-Neu5Ac9Ac^B^ | 1068.5 | 3208.5 | 3207.9 | 1Neu5Ac, 3Hex, 2HexNAc, 2Hep, 2Kdo, 2PE, LipidA-OH |
|  | 1076 | 3231 | 3229.9 | 1Neu5Ac, 3Hex, 2HexNAc, 2Hep, 2Kdo, 2PE, LipidA-OH + Na |
|  |  |  |  |  |
| CMP-Neu5Gc | 1033 | 3102 | 3100.8 | 1Neu5Gc, 3Hex, 2HexNAc, 2Hep, 2Kdo, 1PE, LipidA-OH |
|  | 1073.5 | 3223.5 | 3223.9 | 1Neu5Gc, 3Hex, 2HexNAc, 2Hep, 2Kdo, 2PE, LipidA-OH |
|  | 1081.5 | 3247.5 | 3245.9 | 1Neu5Gc, 3Hex, 2HexNAc, 2Hep, 2Kdo, 2PE, LipidA-OH + Na |
|  |  |  |  |  |
| CMP-Neu5Gc8Me | 1037.5 | 3115.5 | 3114.8 | 1Neu5Gc8Me, 3Hex, 2HexNAc, 2Hep, 2Kdo, 1PE, LipidA-OH |
|  | 1078.5 | 3238.5 | 3237.9 | 1Neu5Gc8Me, 3Hex, 2HexNAc, 2Hep, 2Kdo, 2PE, LipidA-OH |
|  | 1086 | 3261 | 3259.9 | 1Neu5Gc8Me, 3Hex, 2HexNAc, 2Hep, 2Kdo, 2PE, LipidA-OH + Na |
|  |  |  |  |  |
| CMP-Leg5Ac7Ac | 1036 | 3111 | 3109.8 | 1Leg5Ac7Ac, 3Hex, 2HexNAc, 2Hep, 2Kdo, 1PE, LipidA-OH |
|  | 1077 | 3234 | 3232.9 | 1Leg5Ac7Ac, 3Hex, 2HexNAc, 2Hep, 2Kdo, 2PE, LipidA-OH |
|  | 1084 | 3255 | 3254.9 | 1Leg5Ac7Ac, 3Hex, 2HexNAc, 2Hep, 2Kdo, 2PE, LipidA-OH + Na |
|  |  |  |  |  |
| CMP-Neu5Ac9Az | 1036 | 3111 | 3109.8 | 1Neu5Ac9Az, 3Hex, 2HexNAc, 2Hep, 2Kdo, 1PE, LipidA-OH |
|  | 1076.5 | 3232.5 | 3232.9 | 1Neu5Ac9Az, 3Hex, 2HexNAc, 2Hep, 2Kdo, 2PE, LipidA-OH |
|  | 1084 | 3255 | 3254.9 | 1Neu5Ac9Az, 3Hex, 2HexNAc, 2Hep, 2Kdo, 2PE, LipidA-OH + Na |

^A^ Na adducts in solvent

^B^ 9Ac will be lost in the de-O-acylation step
